# Investigating the differential microRNAs expression in young and aged *Drosophila melanogaster* following Flock House Virus infection

**DOI:** 10.1080/21505594.2025.2549497

**Published:** 2025-08-25

**Authors:** Amber Thibeaux, Max Yang Lu, Marshall Martin, Michael Rodwell, Victoria Faber, Lakbira Sheffield, Janna L. Fierst, Stanislava Chtarbanova

**Affiliations:** a Department of Biological Sciences, The University of Alabama, Tuscaloosa, AL, USA; b Department of Biological Sciences, Florida International University, Miami, FL, USA; c Center for Convergent Bioscience & Medicine, University of Alabama, Tuscaloosa, AL, USA; d Alabama Life Research Institute, University of Alabama, Tuscaloosa, AL, USA

**Keywords:** *Drosophila melanogaster*, innate immunity, host–pathogen interactions, aging, micro RNAs, virus infection

## Abstract

MicroRNAs (miRNAs) are small non-coding RNAs ~ 19–22 nt long that post-transcriptionally regulate their mRNA targets. In *Drosophila melanogaster*, the role of miRNAs has mostly been studied in regard to bacterial infection, yielding insights about their regulatory function in innate immunity. However, fewer studies have focused on viral infections, and importantly, how miRNAs modulate aging immune responses is not fully understood. Here, we performed small RNA-sequencing demonstrating that systemic Flock House Virus (FHV) infection of *Oregon-R* flies leads to differential microRNA expression and that this response differs with aging. Gene Ontology and Kyoto Encyclopedia of Genes and Genomes analyses identified cellular pathways and biological processes which may be regulated by dynamic expression of microRNAs during infection. For 17 candidate miRNAs, we tested *Drosophila* lines with *in vivo* miRNA knockdown for their survival of systemic FHV challenge. In response to infection, among miRNA knockdown lines, females consistently outlived males, and young flies generally outlived their aged counterparts. Furthermore, miRNA knockdown lines generally displayed increased susceptibility to viral infection in comparison to controls, particularly prominent among males. For one miRNA chosen for further study, *miR-311*, its dysregulation resulted in decreased survival independent of changes in viral load, suggesting a role in disease tolerance rather than resistance. Lastly, knockdown of the *immune deficiency* (*imd*) gene – a predicted target of *miR-311* - was associated with improved survival of FHV. This work identifies changes in miRNA expression in the aging antiviral response and highlights one miRNA with a role in disease tolerance to FHV in *Drosophila*.

## Introduction

Globally, despite advances in public health such as vaccines, antiviral drugs, and improvements in public health systems and knowledge, viral diseases continue to represent to significant burdens with regard to mortality and morbidity [1]. Proper regulation of immune responses is vital to counteract such infections. While it remains crucial to mount a sufficiently potent immune response, it is also important for organisms to avoid the overactivation of immunity, which leads to detrimental outcomes such as tissue damage [2,3].

Proper regulation of antiviral immunity is especially important for older individuals, who experience increased severity and mortality compared to younger populations [4–6]. Immunosenescence, the deterioration of the immune system caused by age-related alterations in both adaptive and innate immunity pathways, leads to such increased susceptibility to viral infections and decreased vaccine efficacy in older individuals [7]. With an ever-aging population [8], research of the underlying causes of immunosenescence has become increasingly important to improve the health and treatment of that growing population. Given that innate immunity acts as the first line of defense against all immune challenges and is critical for successful clearance of viral pathogens, further exploration into age-associated changes in innate immunity is vital to counteract immunosenescence and improve viral infection outcomes in aged hosts.

The fruit fly *Drosophila melanogaster* serves as an important model for studying immunosenescence. Lacking an adaptive immune system, the use of *Drosophila* allows for isolation of the effects of innate immunity. Due to their short lifespan and development time, flies could be considered “aged” after a relatively short time, and their large number of offspring, low cost of culture, and small, easily manipulated genome further support their use as a model organism.

In *Drosophila*, several cellular processes contribute to antiviral immunity, including phagocytosis, autophagy, and apoptosis [3]. However, the primary mechanism of antiviral defense in *Drosophila* involves the RNA interference (RNAi) pathways [3]. The RNAi pathways comprise the small interfering RNA (siRNA) pathway, piwi-interacting RNA (piRNA), and microRNA (miRNA) pathway. Among these, in *Drosophila*, siRNAs have been most implicated in antiviral immunity [9]. Within the siRNA pathway, recognition of viral double-stranded RNA (dsRNA) by Dicer-2 (Dcr-2) is followed by formation of a complex between Dcr-2 and the accessory protein R2D2 which allows Dicer to cleave the dsRNA into a 21 nt siRNA duplex [10]. Dcr-2 then loads one strand of the duplex into the protein Argonaute (AGO) 2 (AGO2) to form the RNA-induced silencing complex (RISC) which can inhibit viral translation of both DNA and RNA viruses [10].

MicroRNAs (miRNAs), another class of small RNA molecules, have also emerged as a key regulator in *Drosophila* antiviral immunity, exhibiting dynamic expression patterns during infection and post-transcriptionally regulating immune-related gene expression [11–14]. miRNAs are non-coding RNAs ~ 19–22 nt long that regulate gene expression via complementary base pairing between the seed region (nt 2–7) of the miRNA and the 3’ UTR of the target mRNA, which acts to directly cleave the target mRNA or induce its decay [15,16]. In *Drosophila*, this post-transcriptional regulation is achieved through the formation of RISC [17,18], generated by miRNA loading into a member of the AGO family of proteins, primarily AGO1 but also AGO2 [19].

The short length of the miRNA seed sequence allows for a diverse set of interactions between miRNAs and their mRNA targets, with each miRNA possessing the potential to regulate hundreds of transcripts and each gene being capable of being regulated by multiple miRNAs. Additionally, during biogenesis, miRNA precursors are cleaved into two species, the 5p and 3p species depending on whether they are derived from the 5’ or 3’ arms of the precursor, respectively [20,21]. These two species may exhibit co-expression but often also display differential expression patterns [22–25]. Combined with differences in sequences, and thus mRNA targeting [22,26], these properties expand the diversity of miRNA gene regulation.

In *Drosophila,* multiple resources exist to study the role of miRNAs, including mutant lines allowing for the effects of *in vivo* miRNA overexpression (OE), knockout (KO) or knockdown (KD) to be assayed in time and tissue-specific patterns [27–30].

In *Drosophila*, the role of miRNAs in immunity has largely focused on the two evolutionarily conserved Nuclear factor kappa-light-chain-enhancer of activated B cells (NF-κB) pathways, Toll and Immune deficiency (IMD), which function similarly to mammalian Toll-like receptor/interleukin (IL)-1 receptor pathways and tumor necrosis factor receptor (TNFR) pathway, respectively [14,31]. Though these pathways serve roles in antiviral immunity, they are primarily involved in defense against bacterial and fungal pathogens, the context that their miRNA-mediated regulation has mostly been studied in [14,32,33].

The role miRNAs play in viral infection in *Drosophila* remains sparse apart from a few reports studying the role of miRNAs against the DNA virus invertebrate iridescence virus-6 (IIV6) and the picorna-like virus *Drosophila* C Virus (DCV) [11–13,34]. These studies suggest a complex interplay between viruses and the miRNA host response. During IIV6 infection, expression of the antiviral *miR-305-5p* is upregulated to suppress viral replication [13], which is countered by the virus’s 340 R protein through inhibition of *miR-305* silencing activity. Similarly, *miR-8-5p* promotes resistance against DCV through repression of *Jun-related antigen* (*Jra* or *dJun*); however, its downregulation during infection enhances DCV replication [11]. *miR-956-3p* is also downregulated during DCV infection, though unlike *miR-8-5p*, this downregulation serves to inhibit viral replication through derepression of *Sterile alpha and Armadillo motif* (*Sarm* or *Ect4*) [12]. While these reports have shed light on miRNAs with roles in antiviral immunity, studies addressing the function of this class of small, non-coding RNAs in aging immunity are largely unknown.

Here, we used Flock House Virus (FHV) to study how miRNAs may impact the aged innate antiviral response in *Drosophila*. FHV is a non-enveloped, icosahedral virus of the genus Alphanodaviridae which possesses a single-stranded positive-sense RNA genome. We performed small RNA sequencing (sRNA-seq) on FHV infected and control-injected young and aged male flies to identify miRNAs differentially regulated with infection. Cross referencing miRNA target prediction software with prior transcriptomics data obtained from young and aged control- and FHV-infected flies of the same sex and genotype as used here [35], we then performed Gene Ontology (GO) and Kyoto Encyclopedia of Genes and Genomes (KEGG) enrichment analysis to identify biological processes and pathways that potentially experience differential regulation by dynamic expression of miRNAs during infection. Additionally, for 17 miRNAs that were differentially expressed during FHV infection, we assayed survival of their respective miRNA KD lines of FHV infection among age and sex groups. Our analysis revealed that similarly to prior results in wild-type *Oregon-R* flies [35], young flies tended to outlive their aged counterparts and aged females outlive their age-matched male counterparts. Additionally, young miRNA KD females also outlived their age-matched male counterparts, and compared to a genotypical control, miRNA KD lines also tended to exhibit reduced survival with this being more common among male KD lines both young and aged. From the screen of these 17 miRNA KD lines, we identified *miR-311* as a miRNA with strong effects on survival to FHV challenge. We subsequently assayed survival and viral load after FHV infection in *miR-311* KD, deficiency mutants and OE flies, finding that proper regulation of *miR-311* is vital to immunity independent of changes in viral load. For one predicted target of *miR-311* – the gene *immune deficiency* (*imd*) – we show that its knockdown results in improved survival of FHV. Our results suggest that the protective effects of *miR-311* are due to enhanced disease tolerance, whereby the health of the host is improved without alterations in pathogen load, rather than a resistance mechanism which occurs via changes to pathogen clearance [10]. The information from this study helps improve understanding of the role of miRNAs in *Drosophila*’s aging antiviral immunity and hopefully promotes further exploration of how miRNAs impact viral infection outcomes in aged hosts.

## Materials and methods

### Drosophila stocks and handling

All *Drosophila* stocks were raised and maintained on Nutri-Fly® Bloomington formulation food (Genesee Scientific, Cat #: 66–113) at 25°C. *Oregon-R* (BL-2376) flies were obtained from Bloomington *Drosophila* Stock Center (Bloomington, IN). UAS-mCherry-miRNA.sponge lines [36] used to knockdown *miR-10* (BL-61377), *miR-11* (BL-61378), *miR-12* (BL-61379), *miR-13a* (BL-61380), *miR-31a* (BL-61383), *miR-100* (BL-61391), *miR-219* (BL-61400), *miR-284* (BL-61416), *miR-306* (BL-61424), *miR-308* (BL-61425), *miR-311* (BL-61428), *miR-318* (BL-61435), *miR-954* (BL-61440), *miR-965* (BL-61451), *miR-966* (BL-61452), *miR-989* (BL-61473), and *miR-1010* (BL-61493), were obtained from the Bloomington *Drosophila* Stock Center (Bloomington, IN). For generation of knockdown (KD) lines, males from these lines were crossed with virgin, *Wolbachia*-negative female *Act-5c-Gal4/Cyo* (BL-25374) flies. For conditional overexpression (OE) of *miR-311*, virgin female *Act-5c-Gal4/Cyo-tb-RFP; Tub-Gal80*^*ts*^*/TM6B, hu, tb* flies we then crossed with the *UAS-LUC-miR-311* line (BL - 41,163). *UAS-mCherry.scramble.sponge* control line (BL-61501), the *miR 310–313* deficiency line (w*; Df(2 R)d59; Dr^*1*^*/TM6C, Sb*^*1*^, BL-51327) and the *UAS-imd*^*RNAi*^ line (BL-38933) were obtained from the Bloomington *Drosophila* Stock Center (Bloomington, IN). Control *w*^*1118*^ flies were a gift from Dr. John Yoder at The University of Alabama (Tuscaloosa, AL).

For aging experiments, flies were collected at 0–4 days old, anesthetized with CO_2_, separated by sex, and then placed into vials in a 25°C incubator with a controlled 12h/12h dark/light cycle. Flies were flipped every two to 3 days into new vials containing fresh food until the desired age was reached. For conditional *miR-311* overexpression, (*Act-5c-Gal4; Tub-Gal80*^*ts*^* > UAS-Luc-miR-311*) including respective controls (*Act-5c-Gal4; Tub-Gal80*^*ts*^ *>+* and *UAS-Luc-miR-311>+*) crosses were conducted at the permissive temperature of 18°C, resulting progeny collected at 0–4 days old, and subsequently transferred to a restrictive temperature of 29°C.

For small RNA-seq (sRNA-seq), all “young” flies were 4–8 days-old (labeled as 5 days-old) and “aged” flies (labeled as 25 days-old) were 22–26 days-old for non-injected (Ni) cohorts and 24–27 days-old for both Tris- and FHV-injected cohorts. For all other experiments, “young” flies were aged to 3–7 days-old (labeled as 5 days-old) and “aged” flies were 28–32 days-old (labeled 30 days-old). *Wolbachia*-free flies were used in all experiments.

### Flock House virus stock and injections

The Flock House virus (FHV) stock was a kind gift from Dr. Annette Schneemann (Scripps Research Institute, La Jolla, CA). The titer of the stock was determined to be 2.92E + 06 Median Tissue Culture Infectious Dose (TCID_50_)/mL [35]. Viral load measurements and survival assays involving *miR-311* OE flies and respective controls, as well as in experiments shown in Figure 5(C,D, 6) and supplemental Figures S10-S12. were conducted with a separate stock produced in cell culture with a titer of 2.25E + 06 TCID_50_/mL. Briefly, wells of a 6-well cell culture plate were seeded with 1 mL media (Schneider’s *Drosophila* medium supplemented with 10% fetal bovine serum (FBS) and penicillin–streptomycin (1 U/mL and 1 μg/mL, respectively) containing 5x10^5^ low-passaged S1 cells (DGRC Stock 9; https://dgrc.bio.indiana.edu//stock/9; RRID:CVCL_Z231 [37],), and incubated with 10 μL of FHV at 1.5E + 05 TCID_50_/mL for 72 h at room temperature. Cell culture supernatant was then collected by centrifugation (3 min at 1,000 g at 4°C), and passed through a 0.45 μm Costar Spin-X filter microcentrifuge tube (Corning) for 5 min at 9,000 rpm to obtain final stock. TCID_50_/mL was determined as previously described [38] using the Reed–Muench method [39]. Prepared virus stocks were kept frozen at −80°C until used. For viral load measurements and the survival assays involving *miR-284* KD and OE flies and respective controls, a stock of virus with a titer of 2.57E + 06 TCID_50_/mL was used. For all experiments, three replicates of a minimum of 10 flies for each condition were injected per treatment. For all experiments, 4.6 nL of FHV or sterile control solution (filtered cell culture media for the experiments involving *miR-284* KD and OE, and 10 mM Tris-HCL (pH 7.5) for all other experiments) to account for the effect of the injury was injected into the thorax of the fly under the wing using either Drummond’s Nanoject II or Drummond’s Nanoject III apparatus (Drummond Scientific). Sterile control injections were performed prior to FHV injections, and before performing any injection, all materials were sterilized under UV light in the tissue culture hood for 15 minutes. After injections were performed, the materials were UV sterilized again for 15 minutes. Injected flies were placed into a 22°C incubator (or 29°C incubator in the case of the *miR-311* OE line and its respective controls). For sRNA-seq, fifteen living males from each replicate and experimental condition were CO_2_-anesthetized, placed in separate Eppendorf tubes, and then frozen in a −20°C freezer prior to RNA extraction. For survival experiments, the number of living flies was recorded every 24 hours after being placed in the incubator. For virus load experiments, flies were kept in the 22°C (or 29°C incubator for the *miR-311* OE line and controls) for 72 h before being anesthetized and 5 living flies for each condition collected in an Eppendorf tube and frozen at −20°C for later use in RNA extraction.

### Small RNA-sequencing (sRNA-seq)

Total RNA was extracted from three replicates of 15 males for young and aged cohorts of non-injected (Ni), Tris- or FHV-injected flies at 48 h post-injection that were kept at 22°C using the Quick-RNA MiniPrep Kit (Zymo Research). Total RNA was sent to Novogene Co Ltd for sRNA sequencing. Novogene Co., Ltd. performed quality control for sample purity, degradation, contamination, and integrity before library preparation. No ribodepletion step was performed. Samples that passed quality control had sequencing libraries prepared using NEBNext Multiplex Small RNA Library Prep Set for Illumina following the manufacturer’s protocols (NEB, USA). Agilent Bioanalyzer 2100 system using DNA High Sensitivity Chips was used to check library quality (Agilent). Libraries were sequenced using Illumina 50-bp single-end reads. Novogene Co., Ltd performed bioinformatics analysis to map, identify, and determine differentially expressed miRNAs. Novogene Co., Ltd first mapped small RNAs to the reference *Drosophila melanogaster* genome (dmel_r6.23_FB2018_04) using Bowtie [40] with no mismatches. Potential miRNAs were identified using modified mirdeep2 [41] and used miRbase20.0 as the reference for miRNA sequences in *Drosophila melanogaster*. Custom scripts were used to obtain miRNA counts. DESeq R package (1.8.3) was used for differential expression analysis [42], and P-values were adjusted using the Benjamini & Hochberg method. A P-value < 0.05 was used as the cutoff of significance.

### Process and pathway analysis

TargetScanFly Release 7.2 [43], DIANA-micro-T-CDS (employing DIANA’s default interaction score threshold of 0.7) [44], and DIANA-TarBase v8.0 [45] were used to determine the predicted targets for each differentially expressed miRNA in young and aged infected flies. Predicted targets from TargetScanFly with poorly conserved sites were also included. These predicted targets were then cross-refenced with prior transcriptomics data from Sheffield et al. 2021 [35] also derived from young and aged *Oregon-R* flies 48 h post-injection with FHV. Only gene targets which displayed the anticipated inverse patterns of expression from their targeting miRNAs were retained for analysis. The transcriptomics data used only contained genes that were significantly differentially expressed from Tris-injected controls at the 48 h post-infection time point for both young and aged FHV-infected flies. We then separated targets of miRNAs into those targeted by miRNAs upregulated only in young flies, targeted by miRNAs downregulated only in young flies, targeted by miRNAs upregulated only in aged flies, targeted by miRNAs downregulated only in aged flies, targeted by miRNAs upregulated in both young and aged flies, and targeted by miRNAs downregulated in both young and aged flies. We used the Database for Annotation, Visualization, and Integrated Discovery (DAVID) 6.8 [46,47] to analyze for enriched gene ontology (GO) and Kyoto Encyclopedia of Genes and Genomes (KEGG) pathways using a cutoff P-value of 0.1.

### Viral load determination

FHV virus load was determined using reverse transcription quantitative PCR (qRT-PCR) for FHV *RNA1* (*FHV1*). Total RNA was extracted from three replicates of 5 frozen flies per replicate and condition using the Quick-RNA MiniPrep Kit (Zymo Research), according to manufacturer’s instructions. A slight modification included the incubation time after the addition of the DNase treatment being increased from 15 minutes to 20–25 minutes. All samples were eluted into 50 μL of DNase/RNase free water. RNA concentration was determined using NanoDrop One^C^ (Thermo Scientific) and then stored in the freezer at −20°C until used for cDNA synthesis. Next, isolated RNA was converted to cDNA using the High-Capacity cDNA Reverse Transcriptase Kit without RNase inhibitor (Applied Biosystems) using 500 ng of RNA. The reaction was run in the thermal cycler (Eppendorf vapo.protect) following manufacturer’s protocol (25°C for 10 minutes, 37°C for 120 minutes, 85°C for 5 minutes, and then 4°C until cDNA was collected and stored at −20°C). For qRT-PCR, cDNA was diluted 10 times and the PowerUp SybrGreen (Applied Biosystems) master mix used for the reaction. *FHV-1* forward and reverse primers with were used to detect *FHV1*, and *Drosophila*’s *RpL32* was also measured to normalize *FHV-1* expression. See Table S1 for sequences. Relative gene expression was calculated using the formula: 2^(Ct (*RpL32*))^/2^(Ct (*FHV-1*))^. The relative expression value was log-transformed for subsequent statistical analysis.

### Statistical analysis

For all comparisons of survival, statistical significance (P-value) was determined using the Log-Rank Test (Mantel–Cox Test). Statistical analyses were performed using GraphPad Prism Version 9.5.1 for Mac. For all survival analyses: ns = non-significant (*p* > 0.05); * = P < 0.05; ** = P < 0.01; *** = P < 0.001; **** = P < 0.0001. Statistical significance for viral load was determined using a two-way ANOVA test followed by Tukey’s multiple comparisons test. These statistical analyses were also performed using GraphPad Prism. A P-value < 0.05 was considered significant.

## Results

### miRnas are differentially expressed with age and virus infection

To assess miRNA expression changes following FHV infection, we performed sRNA-seq on 5-day old (young) and 25-day old (old) FHV-infected *Oregon-R* male flies at 48 h post-injection. Age-matched males injected with Tris were used as controls to account for miRNA expression changes due to the injection procedure. sRNA-seq was also performed on young and old non-injected (Ni) flies to account for miRNA expression changes due to aging alone in the absence of injury. 48 h post-treatment, six cohorts (young and aged, Ni, Tris- or FHV-injected) with three replicates each of 15 whole flies were collected. Across the 18 samples there were over 620 million total reads, with between 27–62 million reads in each sample (Dataset S1). For all samples, the sequence length distribution for nucleotides 18–28 is shown in (Figure S1). The peak of length distribution was 22 nt followed by 21 nt. There was a total of roughly 603 million clean reads, representing 97.14% of the total reads. The mean Q20, Q30, and GC content values were 96.21%, 93.04%, and 48.16% for samples, respectively (Dataset S1). Over 560 million of these reads were tied to sRNA, with between 23 and 50 million sRNA reads in each sample. The mapping rate values to the *Drosophila* genome among libraries ranged from 70.89% to 93.37% with an average of 81.82% among libraries (Dataset S1). A total of 380 mature and 236 hairpin miRNAs were mapped with the number of mature miRNAs ranging from 230 to 324 among the 18 libraries, and the number of hairpin miRNAs ranging from 168 to 210 among the libraries (Dataset S1). Additionally, there were 10 novel mature and 10 novel hairpin miRNAs predicted among the 18 libraries total (Dataset S1), raw readcounts of miRNA expression are displayed in Dataset S1.

**Figure 1.**
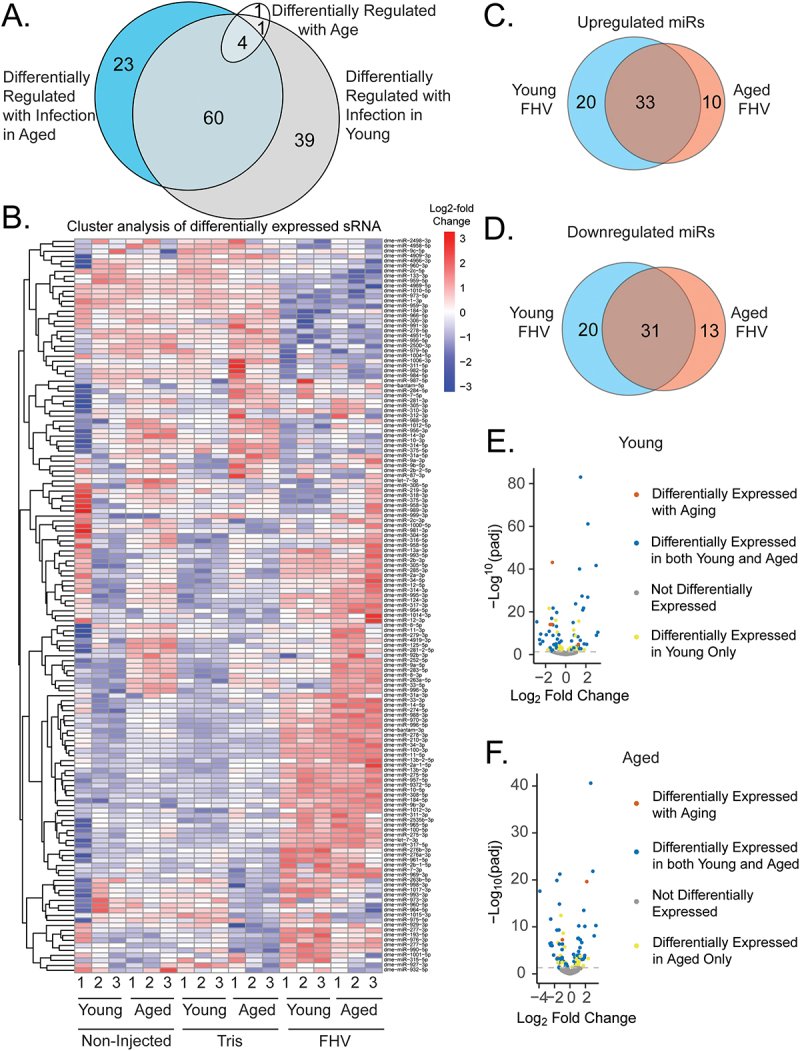
miRNA expression changes 48 post-injection with FHV.

Between young and aged FHV-infected flies, 127 miRNAs displayed differential expression in comparison to age-matched Tris-injected flies, and another was differentially expressed with aging but not infection (Figure 1(A-B) and Dataset 2). With infection, 23 miRNAs were differentially expressed in only aged flies, 40 were differentially expressed in only young flies, and 64 miRNAs displayed common differential expression between groups (Figure 1(A)). In comparison to Tris-injected flies, FHV infection led to differential regulation of a total of 104 and 87 miRNAs in young and aged flies, respectively (Figure 1(A-D)).

The cutoff p-value for significant differential expression was 0.05. (A) Venn diagram displaying the number of miRNAs differentially regulated with aging and infection in young and aged flies (B) Clustered heatmap of miRNA expression 48 h post treatment in young and aged flies that were non-injected, injected with a Tris injury control, or injected with FHV (C) Venn diagram displaying the number of miRNAs upregulated 48 h post-infection with FHV compared to Tris-injected controls among young, aged, or both groups of flies (D) Venn diagram displaying the number of miRNAs downregulated 48 post-infection with FHV compared to Tris-injected controls among young, aged, or both groups of flies. (E) Volcano plot displaying differential miRNA expression 48 h post-infection in young FHV-injected flies compared to young Tris-injected controls. *miR-210-3p*, for which statistical tests regarding differential expression during aging were unable to performed, was excluded from the volcano plots. (F) Volcano plot displaying differential miRNA expression 48 h post-infection in aged FHV-injected flies compared to aged Tris-injected controls.

Of 128 miRNAs that displayed differential expression with infection or aging, 6 were differentially expressed with aging itself in the absence of injury, though these results may have been affected by an outlier amongst one of the young, non-injected samples (Figure 1(B)). Only one miRNA, *miR-4919–3*, was differentially expressed (upregulated) with aging but not during infection. The other 5 miRNAs which were differentially expressed with aging alone were also differentially expressed during infection. *miR-277-5p* was upregulated with infection in both young and aged flies, however, it was downregulated with aging in the absence of injury. *miR-2c-5p*, *miR-1-3p*, and *miR-959-5p* were downregulated with aging alone and also FHV infection in young and aged flies (Figure 1(B-F)). Another miRNA, *miR-33-5p*, displayed greater expression with aging in the absence of injury, but was found to be downregulated with FHV infection only in young flies.

Among the miRNAs found to be differentially expressed with infection, 20 were upregulated and downregulated, respectively, in only young flies (Figure 1(C,D)), and 13 and 10 were upregulated and downregulated in aged flies, respectively. Common to both aged and young flies, after infection 31 miRNAs were upregulated and 33 downregulated as well. Together, these results indicate that FHV infection leads to the significant differential regulation of miRNAs in both young and aged cohorts, and that some miRNAs are specifically regulated across age groups.

### Biological processes associated with metabolism and reproduction are targeted by differentially expressed miRnas

**Figure 2.**
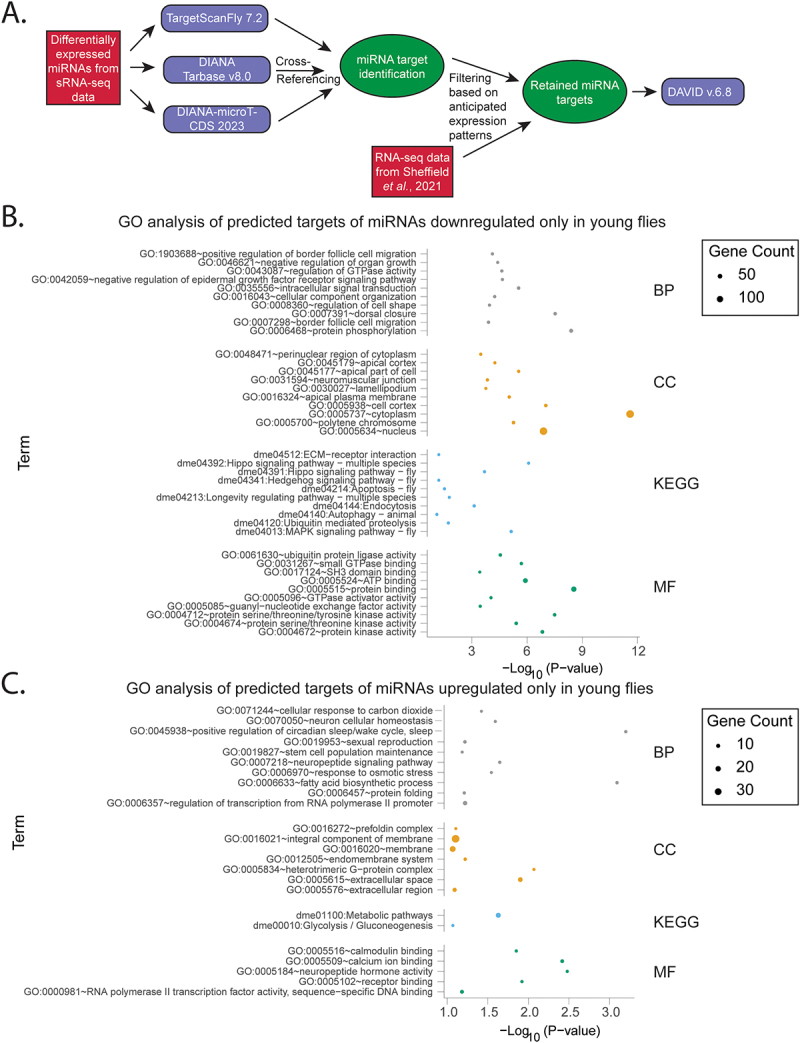
Process and pathway analysis of predicted targets of miRnas differentially expressed with infection in young flies only.

To gain insights about the significance of differential miRNA expression, we evaluated the predicted targets of miRNAs found to be differentially expressed in our sRNA-seq data. This allowed us to also determine how age influenced biological processes regulated by miRNAs in young and aged flies during infection. First, we used TargetScanFly 7.2 [43], DIANA TarBase v8.0 [45] and DIANA-microT-CDS 2023 [44] to obtain targets of all differentially expressed miRNAs. Because there exist high false-positive rates in miRNA target prediction tools, only genes predicted by both TargetScanFly 7.2 and DIANA-microT-CDS 2023 as targets of the differentially expressed miRNAs or which have received experimental support according to DIANA TarBase v8.0 were retained for analysis. To further reduce the prevalence of false positives in our analysis, because miRNAs typically act as repressors of gene expression [16], only genes which display the anticipated inverse patterns of expression from their predicted regulatory miRNAs were retained for GO analysis. For example, for a miRNA upregulated in young flies with infection, only gene targets downregulated in young flies with infection were retained for analysis. Data regarding differential expression of these potential gene targets was derived from Sheffield *et al*. 2021 [35]. These retained targets (filtering schematic is shown in Figure 2(A)) were then analyzed with DAVID v.6.8, performing GO analysis and focusing on enriched biological processes and KEGG pathways. Among the filtered targets, the process and pathway analysis was separated by upregulation/downregulation and the conditions under which the miRNAs were differentially expressed (upregulated in young only, downregulated in young only, upregulated in both young and aged, downregulated in young and aged, upregulated in aged only, downregulated in aged only). The number of miRNAs for each category as well as the number of significantly enriched pathways and processes for each category is displayed in Table 1 and the full list of significantly enriched GO and KEGG terms is in Dataset S3.

(A) Schematic depicting the process for filtering potential *miRNA* gene targets with differential expression during infection for input into Gene Ontology and (GO) Kyoto Encyclopedia Genes and Genomes (KEGG) analysis. The 10 most significantly enriched categories for Gene Ontology Biological Process (BP), Cellular Compartment (CC), Molecular Function (MF) and KEGG analysis are displayed. Pathway and process analysis is divided into (B) predicted targets of miRNAs downregulated only in young flies (C) predicted targets of miRNAs upregulated only in young flies.

**Table 1.** Number of miRnas differentially expressed during infection and number of retained predicted targets, processes and pathways after filtering.

Group of miRNAs	Number of Differentially Expressed miRNAs	Number of predicted gene targets after filtering	Number of significantly enriched GO BP Terms	Number of significantly enriched GO CC Terms	Number of significantly enriched GO MF terms	Number of significantly enriched KEGG terms
miRNAs Downregulated in Young Only	19	448	174	41	50	12
miRNAs Upregulated in Young Only	20	142	15	7	5	2
miRNAs Downregulated in both Young and Aged	30	758	276	73	66	15
miRNAs Upregulated in both Young and Aged	30	326	15	11	10	9
miRNAs Downregulated in Aged Only	10	365	172	37	40	9
miRNAs Upregulated in Aged Only	13	117	14	2	5	4

**Figure 3.**
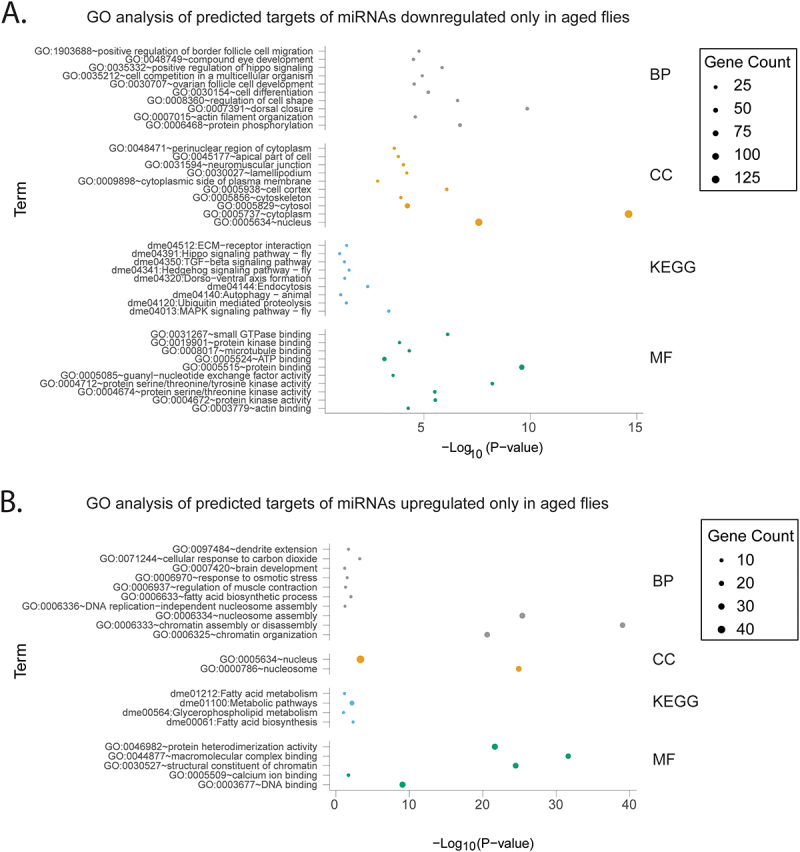
Process and pathway analysis of predicted targets of miRnas differentially expressed with infection in aged flies only.

**Figure 4.**
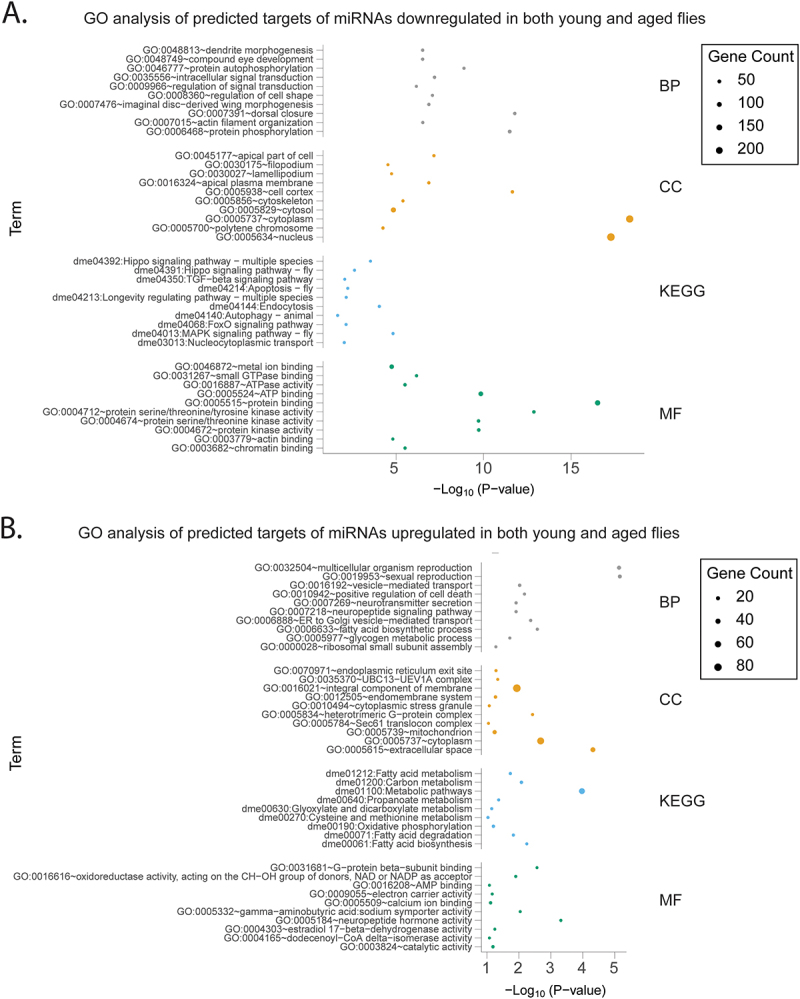
Process and pathway analysis of predicted targets of miRnas differentially expressed with infection in both young and aged flies.

Among the pathway analysis for filtered targets of upregulated miRNAs (downregulated gene targets), the GO Biological Process (BP) the KEGG analysis contained significant enrichment for metabolic terms whether in the analysis of miRNAs upregulated in only young flies (Figure 2(B,C)), upregulated in only aged flies (Figure 3), or that experienced upregulation common to both groups (Figure 4). Among the filtered targets of miRNAs which were upregulated in both young and aged flies the 9 enriched KEGG terms were “metabolic pathways,” “fatty acid biosynthesis,” “carbon metabolism,” “fatty acid degradation,” “fatty acid metabolism,” “propanoate metabolism,” “oxidative phosphorylation,” “glyoxylate and dicarboxylate metabolism,” and “cysteine and methionine metabolism.” For these miRNAs, the GO BP analysis contained similar terms like “fatty acid biosynthetic process” and “glycogen metabolic process.” Similar enrichment was present in miRNAs upregulated either only in young (KEGG terms: “metabolic pathways” and “glycolysis/gluconeogenesis;” GO term: “fatty acid biosynthetic process”) or only in aged flies (KEGG terms: “fatty acid biosynthesis,” “metabolic pathways,” “fatty acid metabolism,” “glycerophospholipid metabolism;” GO terms: “fatty acid biosynthetic process,” “glycogen metabolic process,” and “triglyceride biosynthetic process”). The GO BP term “response to osmotic stress,” associated with infection and implicated in inflammation [48], was also common to all three groups of upregulated miRNAs (miRNAs upregulated in young only, aged only, or in both).

The 10 most significantly enriched categories for Gene Ontology Biological Process (BP), Cellular Compartment (CC), Molecular Function (MF) and Kyoto Encyclopedia of Genes and Genomes (KEGG) analysis are displayed. Pathway and process analysis is divided into (A) predicted targets of miRNAs downregulated only in aged flies (B) predicted targets of miRNAs upregulated only in aged flies

The 10 most significantly enriched categories for Gene Ontology Biological Process (BP), Cellular Compartment (CC), Molecular Function (MF) and Kyoto Encyclopedia of Genes and Genomes (KEGG) analysis are displayed. Pathway and process analysis is divided into (A) predicted targets of miRNAs downregulated in both young and aged flies (B) predicted targets of miRNAs upregulated in both young and aged flies

In analyzing filtered predicted targets of miRNAs upregulated in both young and aged flies, “sexual reproduction” was among the most significantly enriched GO BP terms. Among miRNAs upregulated only in young flies, this term is present along with the term “mating behavior, sex discrimination.” However, reproduction-related terms are absent among the GO BP terms for miRNAs upregulated in only in aged flies. If this pathway analysis is representative of actual differential repression of reproductive processes between young and aged flies, this could mediate survival differences to FHV between age groups [35] given the well-established trade-offs between immunity and reproduction [49,50].

Among the pathway analysis of filtered targets of downregulated miRNAs (upregulated gene targets) whether downregulated in young only (Figure 2), aged only (Figure 3), or both (Figure 4), there was significant enrichment of conserved signaling pathways. Common to all three groups were the KEGG terms “MAPK signaling pathway – fly” and “Hippo signaling pathway – fly” and related GO terms “positive regulation of Hippo signaling.” The KEGG term “TGF-beta signaling pathway” was also common to both miRNAs downregulated in only aged or both young and aged flies but was not significantly enriched among terms for miRNAs downregulated only in young flies.

The stress response KEGG terms “ubiquitin mediated proteolysis” and “autophagy – animal” (along with corresponding GO BP term “autophagy”) were common to miRNAs downregulated in young only, aged only, or in both age groups (Figure 2–4). Additionally, the KEGG term “apoptosis – fly” was only found for miRNAs downregulated in young (Figure 2) and miRNAs downregulated only in both young and aged flies (Figure 4).

Among the filtered targets of miRNAs which were downregulated in both young and aged flies (upregulated gene targets), unsurprisingly, many immune or stress response-related terms were prominent including “regulation of defense response to virus,” “JAK-STAT cascade,” “stress granule assembly,” “regulation of JNK cascade,” “hemocyte migration” (a class of *Drosophila* immune, macrophage-like cells [51]), “RNA interference,” “defense response to virus, “cellular response to hypoxia,” “response to oxidative stress,” and “JNK cascade.” Collectively, the process and pathway analysis suggest that in addition to genes involved in metabolism and reproduction, miRNAs may target genes known to function in immunity.

### In vivo miRNA knockdown leads to increased susceptibility of FHV infections and is age- and sex-dependent

Because miRNAs can regulate many different transcripts, including those which play roles in antiviral immunity, it was anticipated some miRNAs differentially expressed with infection would lead to altered virus susceptibility when knocked down. To test this, for several miRNAs with at least a significant 2-fold change in expression during FHV infection in either young, aged, or both groups were ubiquitously knocked down by overexpressing miRNAs sponges using the Gal4/UAS system to create a partial loss of function phenotype for the miRNA [36], with an *Act-5c-Gal4>+* genotypical control (Figures S2-S8 and Tables S2-S4). For these initial screening experiments, survival was assayed in young flies reared to 5 days-old and aged flies reared to 30 days-old which were injected with FHV. Subsequent *in vivo* validation and analysis of candidate miRNAs also included a control Tris buffer injection.

An age-dependent increase in susceptibility to FHV for male *Drosophila* was present in miRNA KD lines, mirroring previous studies in *wild-type Oregon-R* young and aged males [35]. For 14 out of 17 miRNA KD lines, young males outlived aged males with the other 3 miRNA KD lines exhibiting no significant difference (Table 2). While for some miRNA KD lines, females also displayed greater susceptibility to FHV infection with age, this was far less prominent than for males, with just 6 out of 17 aged female miRNA KD fly lines displaying significantly decreased survival compared to their young counterparts. Young males also outlived aged males among the *Act-5c-Gal4>+* controls (*p* = 0.0002); however, for 12 of 17 miRNA KD lines, the age-dependent susceptibility to FHV infection was significantly higher compared to *Act-5c-Gal4>+* controls.

**Table 2.** Survival differences among young and aged miRNA KD flies to FHV infection.

Group	Aged Outlived Young	Non-significant difference	Young outlived Aged
Males	None	*miR-989*, *miR-318*, *miR-11*	*Act-5c-Gal4* Control, *miR-311*, *miR-31a*, *miR-13a*, *miR-219*, *miR-12*, *miR-954*, *miR-965*, *miR-306*, *miR-284*, *miR-10*, *miR-308*, *miR-100*, *miR-1010*, *miR-966*
Females	*miR-13a*, *miR-318*, *miR-10*	*Act-5c-Gal4>+* Control, *miR-31a*, *miR-989*, *miR-954*, *miR-306*, *miR-284*, *miR-11*, *miR-1010*	*miR-311*, *miR-219*, *miR-12*, *miR-965*, *miR-100*, *miR-966*

” >Young miRNA KD flies tend to exhibit enhanced survival to FHV infection compared to their aged counterparts, especially amongst male miRNA KD flies. Statistical significance was determined using the Log-Rank Test (Mantel–Cox Test) with a cut off value of *p* < 0.05 for statistical significance.

Direct sex differences in survival were also prominent, as for every miRNA KD line tested, females outlived age-matched males after FHV infection (Table 3). *Act-5c-Gal4>+* female controls outlived age matched males for both age groups, but young males were not significantly susceptible compared to young females (*p* = 0.144).

**Table 3.** Survival differences among male and female miRNA KD flies to FHV infection.

Group	Male outlived Female	Non-significant difference	Female Outlived Male
Young	None	None	*Act-5c-Gal4* Control, *miR-311*, *miR-31a*, *miR-13a*, *miR-989*, *miR-219*, *miR-318*, *miR-12*, *miR-954*, *miR-965*, *miR-306*, *miR-284*, *miR-10*, *miR-308*, *miR-100*, *miR-11*, *miR-1010*, *miR-966*
Aged	None	None	*Act-5c-Gal4* Control, *miR-311*, *miR-31a*, *miR-13a*, *miR-989*, *miR-219*, *miR-318*, *miR-12*, *miR-954*, *miR-965*, *miR-306*, *miR-284*, *miR-10*, *miR-308*, *miR-100*, *miR-11*, *miR-1010*, *miR-966*

” >All female miRNA KD lines exhibit enhanced survival to FHV infection compared to their age-matched counterparts. Statistical significance was determined using the Log-Rank Test (Mantel-Cox Test) with a cut off value of *p* < 0.05 for statistical significance.

Additionally, compared to the *Act-5c-Gal4>+* control, 16 out of 17 lines displayed increased susceptibility to infection among both young and aged males, compared to just 9 and 12 lines for young and aged females, respectively (Table 4). Our analysis indicates that the effect of miRNA KD on survival of FHV infection differs between males and females. Furthermore, male *Drosophila* displays a significant age-dependent increase in susceptibility to FHV with miRNA KD compared to females.

**Table 4.** Survival differences between miRNA KD flies and *act-5c-Gal4>+* control to FHV infection.

Group	Control Outlived miRNA KD	Non-significant difference	miRNA KD Outlived Control
Young Males	*miR-311*, *miR-31a*, *miR-13a*, *miR-989*, *miR-219*, *miR-318*, *miR-12*, *miR-965*, *miR-306*, *miR-284*, *miR-10*, *miR-308*, *miR-100*, *miR-11*, *miR-1010*, *miR-966*	*miR-954*	None
Aged Males	*miR-311*, *miR-31a*, *miR-13a*, *miR-989*, *miR-219*, *miR-12*, *miR-954*, *miR-965*, *miR-306*, *miR-284*, *miR-10*, *miR-308*, *miR-100*, *miR-11*, *miR-1010*, *miR-966*	*miR-318*	None
Young Females	*miR-13a*, *miR-989*, *miR-318*, *miR-306*, *miR-10*, *miR-308*, *miR-11*, *miR-1010*, *miR-966*	*miR-311*, *miR-31a*, *miR-12*, *miR-284*, *miR-100*	*miR-219*, *miR-954*, *miR-965*
Aged Females	*miR-311*, *miR-989*, *miR-219*, *miR-12*, *miR-965*, *miR-306*, *miR-284*, *miR-308*, *miR-100*, *miR-11*, *miR-1010*, *miR-966*	*miR-31a*, *miR-13a*, *miR-318*, *miR-10*	*miR-954*

” >miRNA KD in flies tends to impair survival to FHV infection compared to the *Act-5c-Gal4>+* genotypical control, which is particularly prominent among young and aged male miRNA KD lines. Statistical significance was determined using the Log-Rank Test (Mantel–Cox Test) with a cut off value of *p* < 0.05 for statistical significance.

### miR-311 dysregulation increases susceptibility to FHV in Drosophila independently of virus load

**Figure 5.**
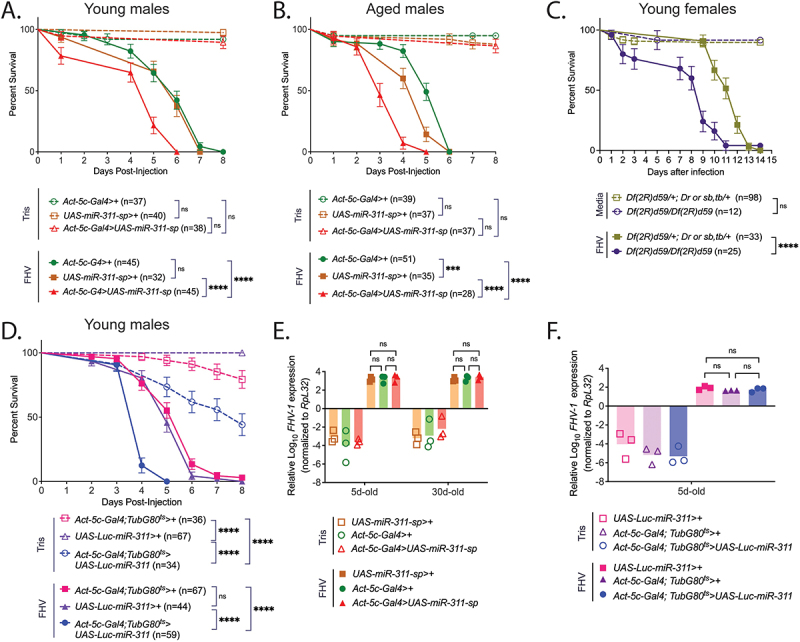
*miR-311* dysregulation increases susceptibility to FHV infection but is not associated with changes in viral load.

We identified *miR-311* (expression changes shown in Figure S9) as a candidate line chosen for further study given the significant decrease in male survival compared to the *Act-5c-Gal4>+* control especially among aged males (*p* < 0.0001) (Table S4). Young *UAS-miR-311-sp>+* flies did not display significant differences in survival compared to the young *Act-5c-Gal4>+* control (*p* = 0.7156). However, young *miR-311* KD flies exhibited increased susceptibility compared to both the young *UAS-miR-311-sp>+* control (*p* < 0.0001) and *Act-5c-Gal4>+* control (*p* < 0.0001) (Figure 5(A)). Survival proportions for 30-day KD males were 46.43% at 3 days post-infection compared to 88.24% for *Act-5c-G4>+* and 88.6% for *UAS-miR-311-sp>+* controls. 30-day *UAS-miR-311-sp*>+ males died faster compared to *Act-5c-Gal4>+* aged males (*p* = 0.0009), while aged *miR-311* KD males remained more susceptible to FHV than aged *UAS-miR-311-sp>+* males (*p* < 0.0001) (Figure 5(B)). Comparing the survival of FHV of young *miR-311* KD to a line overexpressing a scrambled miR sponge (*Act-5c-Gal4 > UAS-mCherry.scramble-sp*; control), we observed that male *miR-311* KD flies outlived the controls (median survival of 10 days and 9 days, respectively; *p* < 0.0001), while females *miR-311* KD succumbed faster (median survival of 10d and 11.5d, respectively; *p* < 0.0001) (Figure S10). In aged flies, however, *miR-311* KD of both sexes died significantly faster than the miR scramble controls (males: median survival of 7 days and 8 days, respectively; *p* < 0.0001; females: median survival of 9 days and 11 days, respectively; *p* < 0.0001) (Figure S10). We also tested how in comparison to heterozygous controls, FHV infection affected the survival of homozygous flies carrying a deletion spanning *miR-310*- *miR-313* that also removed *miR-311* (Figure 5(C)). Due to the deficiency stock being generally unhealthy and the inability to collect large homozygous progeny, we were able to only test 5-day-old homozygous females. In comparison to heterozygous controls, deficiency homozygotes died significantly faster of FHV (*p* < 0.0001), while the survival curves in response to control media injection were comparable between the two experimental cohorts (*p* = 0.8206) and did not result in increased mortality (Figure 5(C)).

(A) *miR-311* knockdown increases susceptibility to FHV in young males (B) *miR-311* knockdown increases susceptibility to FHV in aged males (C) Homozygous young female flies carrying a deficiency that includes *miR-311* (*Df(2 R)d59*) display increased susceptibility of FHV in comparison to heterozygous controls (D) *miR-311* overexpression increases susceptibility to FHV in young males. Statistical significance was determined using the Log-Rank Test (Mantel–Cox Test) wherein ns = not significant (*p* > 0.05); * = P < 0.05; ** = P < 0.01; *** = P < 0.001; **** = P < 0.0001. (E) *miR-311* knockdown in young and aged flies is not associated with changes in viral load following FHV infection (F) *miR-311* overexpression in young flies is not associated with changes in viral load following FHV infection. Statistical test: two-way ANOVA ith ns = not significant (*p* > 0.05)

We next sought to measure survival outcomes from FHV and Tris injections in flies overexpressing (OE) *miR-311*. However, while attempting to rear *Act-5c-Gal4 > UAS-Luc-miR-311* flies, we encountered a developmental lethal phenotype with constitutive *miR-311* OE. To prevent this lethality, we instead employed a conditional OE specifically in adults using a line that carries Gal4 in combination with a temperature sensitive Gal80 (Gal80^ts^), a Gal4 repressor (*Act-5c-Gal4; Tub-Gal80*^*ts*^). At the permissive temperature (18°C), Gal80 can repress Gal4, while at the restrictive temperature (29°C), Gal80 does not function and Gal4 is able to drive gene expression by binding to a UAS promoter [30].

Though rearing the flies at 18°C and employing temperature-sensitive *miR-311* OE led to eclosion of adults (Table S5), the *miR-311* OE flies remained unhealthy with a short lifespan. When the flies were switched to a 29°C environment to activate OE of *miR-311* after eclosion and beginning at 2-days old, all flies succumbed within ~12 days and thus survival and viral load experiments were only conducted with 5-day-old young flies. To enhance the resolution of survival differences, we used an FHV stock with a lower titer of 2.25E + 05 TCID_50_/mL.

Interestingly, similar to the *miR-311* KD line, *miR-311* OE flies also displayed reduced survival compared to its respective controls *Act-5c-Gal4; TubGal80*^*ts*^*>+* (*p* < 0.0001) and *UAS-Luc-miR-311>+* (*p* < 0.0001) (Figure 5(D)). While this may have been in-part due to the effects of reduced lifespan caused by OE of *miR-311*, such effects were likely limited as survival proportions at 5 days post-infection were 6.77% for *miR-311* OE flies and 73.53% for Tris-injected *miR-311* OE flies. At 6 days post-infection when all FHV-injected *miR-311* OE flies had died, 61.76% of Tris-injected *miR-311* OE flies remained alive. Taken together, our results indicate that *miR-311* dysregulation, whether OE or KD, proves detrimental to survival following FHV challenge.

In parallel of the survival assays, we measured viral loads to examine whether the increased susceptibility resulting from *miR-311* KD and OE was due to compromised disease tolerance or resistance mechanism. We quantified *FHV-1* expression in young and aged *miR-311* KD and young *miR-311* OE flies, as well as age-matched genotypical controls. This was done in flies injected with FHV with a lower titer of 2.25E + 05 TCID_50_/mL to reduce survivorship bias. Because young female *miR-311* KD flies did not display significant differences in susceptibility compared to *Act-5c-Gal4>+* flies (*p* = 0.3748) (Table 4 and Table S4), and in relation to their *Act-5c-Gal4>+* controls, aged females possessed just a modest increase in susceptibility to FHV infection (*p* = 0.0079) compared to aged males (*p* < 0.0001) (Table 4 and Table S4), viral load was only measured in male flies 3 days post-infection.

In-line with a prior report showing that increased mortality of FHV with age is associated with impaired disease tolerance rather than resistance [35], our results did not identify significant differences in viral load between young and aged *miR-311* KD FHV-infected flies (*p* > 0.9999) nor were there any significant differences in viral load between *miR-311* KD and *miR-311* OE flies with their respective genotypical controls (Figure 5(E,F)). Altogether, these results suggest that *miR-311* regulation likely functions as part of disease tolerance rather than resistance in response to FHV infection.

During the screen of 17 miRNA KD lines, *miR-284-5p* also emerged as a miRNA of interest due to its high readcount in sRNA-seq (Dataset S2) as well as the fact that KD decreased survival of FHV in young males and both aged males and females compared to the *Act-5c-Gal4>+* control (Table S4). This suggested it could act as a positive regulator of immunity, and when combined with the fact that it was downregulated only in aged flies (Figure S11A), we speculated it may contribute to impaired survival outcomes in aged flies. We carried additional survival assays with both young and aged male flies and observed that in comparison to genotypic controls (*Act-5c-G4>+* and *UAS-Luc-miR-284>+*, respectively) ubiquitous *miR-284* OE resulted in significant protection of FHV infection in young (*p* < 0.0001and *p* = 0.0252, respectively) but not in aged males which died faster (*p* = 0.0001and *p* = 0.3265, respectively) (Figure S11B, C).

### Knockdown of imd, a predicted target of miR-311, results in improved survival of FHV

**Figure 6.**
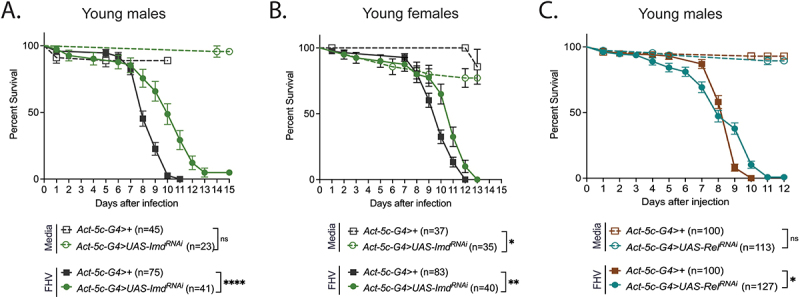
Knockdown of IMD pathway components improves survival of FHV in young flies.

While *miR-311* is predicted to have many gene targets (Table S6), one of them: *immune deficiency* (*imd*) is a known positive regulator of *Drosophila* immunity and the fly’s immune deficiency (IMD) NF-kB pathway [31]. The *miR-310–313* cluster which includes *miR-311* negatively regulates the fly IMD pathway following Gram-negative bacterial infection, and *miR-311* can bind to the 3’UTR of the *imd* gene *in vitro* [52]. Furthermore, in our previous work, we found *imd* to be significantly upregulated following FHV infection and to a greater extent in aged flies [35]. We therefore investigated how ubiquitous downregulation of *imd*, and another component of the IMD pathway, the transcription factor *Relish* (*Rel*), affected survival of FHV infection. We found that in comparison to *Act-5c-G4>+* controls, *imd* knockdown resulted in significantly increased survival of FHV infection in both male (*p* < 0.0001) and female (*p* = 0.0013) young flies (Figure 6(A,B)). Young males with *Rel* knockdown also displayed a slightly improved survival of FHV in comparison to controls (endpoint survival of 12 days vs 10 days for controls, *p* = 0.0426) (Figure 6(C)). However, young females, as well as aged flies of both sexes were more susceptible to FHV than controls (*p* < 0.0001 (young females), *p* = 0.0002 (aged males) and *p* < 0.0001 (aged females)) (Figure S12). This could be due to sex- and age-dependent increase in secondary bacterial infections as IMD pathway knockdown or mutation renders flies highly immunocompromised [53]. Together, these results indicate that in response to FHV infection, *miR-311* may be limiting the activation of the *Drosophila* IMD pathway possibly by directly targeting *imd*.

*imd* KD significantly increases the survival of FHV infection in young (5d) male (A) and female (B) flies. (C) Relish KD significantly improves survival of FHV infection in young (5d) males. Statistical significance was determined using the Log-Rank Test (Mantel-Cox Test) wherein ns = not significant (*p* > 0.05); * = P < 0.05; ** = P < 0.01; *** = P < 0.001; **** = P < 0.0001

## Discussion

In this study, we found that young and aged *Drosophila* systemically infected with FHV have distinct miRNA expression profiles. While this work represents the first instance of sRNA-seq and miRNA analysis following FHV infection in aging *Drosophila*, our data is partly limited by the whole fly sequencing approach. It is possible that at the tissue or organ level, differential miRNA expression may be masked by contrasting expression in other tissues. This could be significant with regard to immunity where this masked differential miRNA expression could occur in an organ such as the fat body (equivalent to the mammalian liver and adipose tissue) which possesses a major role in immunity [31]. Additionally, our whole fly sequencing approach limited the ability to study exosomal miRNAs, wherein certain miRNAs are loaded into exosomes which travel through the circulatory system to target distant cells and tissues [54]. Future studies in the *Drosophila* – FHV aged host-virus model could address these points. This sRNA-seq did predict 10 novel mature miRNAs and 10 novel hairpin miRNAs, which could be validated through cross-referencing with prior datasets or experimental techniques such as qRT-PCR [55].

Here, we found young flies to display a greater number of differentially regulated miRNAs in terms of both down- and upregulation. Our prior transcriptomics data from young and aged flies (young and aged flies were 7d/25d instead of 5d/25d in the present study) 48 h post-infection in the same *Oregon-R* line demonstrated that aged flies exhibited more differential gene expression than their younger counterparts [35]. This leads us to speculate that in young flies, the large number of differentially regulated miRNAs may contribute to stabilization of gene expression, which could potentially represent a source of variation in the efficacy of antiviral responses between young and aged flies. Additional experiments quantifying both miRNA and gene expression at a broader set of time points after FHV infection could aid in providing more conclusive evidence.

In investigating the role of miRNAs in *Drosophila*’s antibacterial responses, quantification of both miRNA expression and of gene targets throughout a diverse set of time points has suggested an overarching role for miRNAs in preventing immune overaction later in the course of infection [14]. Li et al.’s examination of temporal patterns of expression for *miR-277* along with *imd* and *Tab2*, genes which it was predicted to downregulate, has helped yield insights about *miR-277*’s ability to restore immune homeostasis and prevent immune overactivation through targeting of *imd* and *Tab2* [56]. Examination of miRNA expression patterns during *M. luteus* infection in *Drosophila* has also aided in yielding a model in which transcriptional changes of miRNAs later in the immune response act to maintain immune homeostasis and prevent immune overaction [57].

However, when examining data surrounding miRNA expression, it is important to consider that miRNA transcriptional changes may not entirely be an effect of the host immune response, but also pathogen alteration of the host’s immune response as well. In *Drosophila*, the downregulation of *miR-8-5p* during DCV infection has been shown to promote viral accumulation [11], and in mammals, viruses directly downregulate the antiviral *miR-27* [58–61].

Additionally, it is important to consider that transcriptional changes in miRNA expression may not directly correlate with the degree of target repression. Reports have demonstrated that with aging, loading of certain miRNAs into both AGO1 and AGO2 may be affected [62,63], suggesting that transcript abundance is not a perfect readout of miRNA targeting activity. Nonetheless, going forward, similar experiments quantifying miRNA and gene expression at a wider range of time points following viral infection could prove insightful.

We also conducted pathway analysis based on predicted targets of differentially expressed miRNAs with age and infection. However, when examining the predicted pathways and processes, it is important to note that they do not conclusively represent the targeting of miRNAs, as *in silico* target predictions are prone to false positives [64]. However, by cross-referencing multiple target prediction software and setting thresholds for minimum prediction scores along with correlation with transcriptomics data, this analysis provides a rudimentary outline of the processes which miRNAs may be involved in during FHV infection, balancing both the prevalence of false-positive and false-negative predictions in our analysis. It is also important to consider that this analysis does not capture the degree to which each pathway is repressed or upregulated.

In our study, common to potential gene targets of all groups of downregulated miRNAs (downregulated in young or aged only, or both), GO and KEGG analysis identified conserved and broadly functioning pathways that have been implicated in *Drosophila* immunity such as the Hippo and Mitogen Activated Protein Kinase (MAPK) pathways (Figure 2–4). The Hippo pathway is primarily noted for its involvement in tissue regeneration and cell proliferation but also is implicated in Toll pathway regulation [65]. KD of Hippo pathway components in *Drosophila* increases susceptibility to fungal and gram-positive bacterial infection (pathogens that trigger Toll pathway activation) but not gram-negative bacteria (pathogens activating the IMD pathway). Conversely, in mammalian models, the Hippo pathway acts as a negative regulator of antiviral immunity [66]. Similarly, the conserved MAPK pathway, canonically involved in tissue homeostasis and stress responses [67], has also been implicated as a negative regulator of IMD/NF-κB signaling in *Drosophila* [68]. Additionally, the TGF-β pathway, enriched for miRNAs downregulated in only aged or both young and aged flies, has a suggested role in immunity to suppress immune overaction [69].

Unsurprisingly, among filtered targets of miRNAs downregulated in both aged and young flies, there was also enrichment for processes such as the JNK and JAK-STAT cascades, RNAi, and other stress-related processes such as “cellular response to hypoxia” and “response to oxidative stress” (Figure 4 and Dataset S3). In *Drosophila*, both the JNK cascade, a part of the MAPK pathway, and JAK-STAT cascades have demonstrated roles in immunity [51]. RNAi also represents the main mechanism of antiviral immunity in *Drosophila* [3] and responses to hypoxia and oxidative stress are other processes with demonstrated immune roles in a multiple contexts [48].

In our screen of 17 miRNA KD lines, we uncovered significant sexual dimorphism, with all female lines outliving aged-matched males (Table 3 and Table S3). Our prior work employing *wild-type Oregon-*R flies also revealed that aged females outlived aged males after FHV infection [35], and it is possible that genetic-background differences independent of miRNA KD may to some degree mediate some of these sexually dimorphic immune phenotypes. However, miRNA expression differences may also play a role, as sex-biased expression of miRNA has already been uncovered in the absence of infection [70]. Follow-up experiments performing sRNA-seq in female flies could help provide insight regarding whether miRNA expression differences during infection contribute to sexually dimorphic immune phenotypes observed in our prior work [35].

However, whereas those prior results in *Oregon-R* flies demonstrated survival differences between sexes for aged but not young flies, among miRNA KD lines, we found compared to *Act-5c-Gal4>+* controls, miRNA KD increased the significance by which young females outlived young males for all lines tested (Table S3). Additionally, miRNA KD increased susceptibility compared to the *Act-5c-Gal4>+* control in 16 out of 17 lines for young and aged males, compared to just 9 and 12 lines for young and aged females, respectively (Table 4). These results suggest that miRNA KD exacerbates sexual dimorphism of immune phenotypes beyond genetic-background differences, although the mechanism by which this is accomplished is not immediately apparent.

Further analysis of miRNA expression is needed to determine if there exist differences in miRNA KD efficacy between sexes that could mediate such differences. Another potential factor is miRNA loading efficacy. One report by Gartland et al. 2022 demonstrated differential loading of *miR-34-5p* between aged females and males [62], and we cannot rule out the possibility that sex-differences in miRNA loading occur in both young and aged flies after miRNA KD.

As another element of sexual dimorphism among miRNA KD lines, we found that similarly to *Oregon-R* flies [35], among the miRNA KD lines, 30-day-old males tended to have significantly increased susceptibility to FHV, while altered susceptibility among age-matched female miRNA KD flies was far more inconsistent with either no significant differences or aged females outliving younger counterparts in many instances. Similarly to other age and sex effects observed, genetic background differences with aging, or differences in KD efficacy or miRNA loading efficacy may be involved in these survival patterns.

We selected *miR-311* as a candidate miRNA for further studies. We measured survival and virus load in *miR-311* KD or OE flies and their respective genotypical controls. We also compared survival of FHV in a fly line carrying a homozygous deletion of the *miR-310–313* cluster that also encompasses *miR-311* gene to a heterozygous control. These experiments suggested that dysregulation of *miR-311*, either its OE or KD, impairs survival to FHV likely through a disease tolerance mechanism. *miR-311* has over 400 predicted targets (Table S6), and it is possible that both KD and OE of this microRNA result in a similar phenotype because of target gene deregulation. Our results suggest that proper levels of expression of *miR-311* are needed to ensure adequate responses following FHV infection. Future functional gene studies could address how expression levels of individual *miR-311* targets influence survival outcomes of virus infection. Here, we also did not rule out the possibility that changes in resistance do not occur at the tissue level, as our qRT-PCR was performed in whole flies. Additional viral load and quantification at the tissue or organ level could rule out this possibility and experiments employing other UAS/Gal4 driver lines to induce tissue-specific OE and KD of *miR-311* could identify the tissues within which *miR-311* dysregulation has the greatest effect.

Interestingly, while studies on host–pathogen interactions surrounding pathogen resistance are relatively abundant, mechanisms of disease tolerance remain poorly characterized [10]. It is generally understood that tissue damage control and the stress and damage responses that govern this are the main mechanism of tolerance [48,71], but investigating *miR-311* targets in *Drosophila* could serve as another valuable starting point to identify genes which contribute to survival differences without corresponding differences in viral load. Beginning with *in silico* predicted *miR-311* targets (Table S6), subsequent *in vitro* luciferase reporter assays could be conducted to confirm gene targeting [72]. Additionally, employing fly mutants with overexpression or deficiency of *miR-311* gene targets confirmed *in vivo*, and conducting further survival and virus load assays would indicate whether they mediate the effects of *miR-311* KD or OE based on whether they phenocopy the outcome of impaired survival via a mechanism of disease tolerance. Similar lines of investigation regarding the other miRNAs studied in our screen could further yield insights on other mechanisms of either tolerance or resistance.

When considering potential gene targets that may mediate the immune phenotypes of the *miR-311* KD and OE lines, there may exist some confounding factors as it is not fully understood how artificial miRNA OE and sponge OE (to induce miRNA KD) constructs affect the targeting of the 3p vs 5p species of miRNA. However, in this instance, it is most likely that such effects are primarily attributable to *miR-311-3p*. Prior work in *Drosophila* has demonstrated that biogenesis of the 3p and 5p species occur at similar rates, suggesting differences in transcript abundance are the resulting of differences in half-life [73]. The relatively low abundance of *miR-311-5p* compared *miR-311-3p* in our sRNA-seq especially with infection (Figure S9) likely indicates a shorter half-life of *miR-311-5p*. This lesser transcript abundance and shorter half-life of *miR-311-5p* suggest that *miR-311-3p* is the primary species mediating immune phenotypes in not only the *miR-311* OE survival assays, but also the KD experiments. The lower baseline expression of *miR-311-5p* compared *miR-311-3p* in *wild-type* flies likely also corresponds to a lower magnitude of target gene repression. Given the potentially less significant basal effects of *miR-311-5p*, within the *miR-311* KD flies compared to *wild-type* flies, targeting activities of the more abundant *miR-311-3p* are likely to be more disproportionately disrupted and to mediate phenotypical differences compared to controls.

Among the potential targets of *miR-311-3p* predicted by both DIANA and Targetscan (Table S6), many have established roles in various aspects of *Drosophila* innate immunity or immune-related processes. Though the Toll and IMD pathways are primarily noted for their roles in defense against fungi and bacteria, they have been implicated in antiviral immunity [3]. When considering their potential roles in mediating the immune effects of *miR-311* KD or OE, however, it is important to note that in certain contexts, genes in these defense pathways may serve pro-immune functions while in others they lead to overactivity of the immune system which can impair disease tolerance [3,71].

The *miR-311-3p* target, *Leucine-rich repeat* (*LRR*), has been implicated in Toll pathway-mediated immunity, as its KD promoted upregulation of the Toll pathway antimicrobial products Defensin and Drosomycin [74]. The gene *immune deficiency* (*imd*), a central positive regulator of the IMD pathway [75], is another predicted *miR-311-3p* target, which we tested in this study (Figures 6 and Figure S12). Our previous transcriptomics study showed that *imd* expression was significantly increased 48 h after FHV infection, and to a greater extent in older flies [35]. This is consistent with *miR-311-3p* expression being significantly induced in young but not aged FHV-infected flies (Figure S9). Furthermore, the protective effect for *imd* KD after FHV infection is also consistent with the increased susceptibility of *miR-311* KD flies. Indeed, *miR-311* KD could lead to upregulation of *imd* expression, which in turn could result in immunopathology causing more rapid mortality independently of virus load. A role for IMD pathway-mediated immunopathology is also supported by our results showing that *Rel* (NF-kB) KD in young males improves survival outcomes of FHV infection. Additionally, *in vivo* expression of Attacin, an antimicrobial peptide which primarily defends against gram-negative bacteria [14], was found to be downregulated with RNAi of the predicted *miR-311-3p* target *kayak* (*kay*), a transcription factor involved in the JNK pathway. The JNK pathway is a stress response pathway [76] which has also been implicated in antimicrobial peptide gene expression in another report *in vitro* [77]. Terms surrounding the JNK pathway were also significantly enriched in our process and pathway analysis of the many groups of downregulated miRNAs (Dataset S3).

Other genes involved in the JNK pathway include the positive regulators *ced-12* [78], *hemipterous* (*hep*) [79], *slipper* (*slpr*) [80,81], and the negative regulator *raw* [82,83], all of which are also predicted *miR-311-3p* targets.

One process in which the JNK pathway affects that itself has been implicated in tolerance mechanisms is autophagy [76]. This process was found to be commonly enriched in our process and pathway analysis among all three groups of upregulated miRNAs (Dataset S3). In mouse models, autophagy promotes protection against *Staphylococcus aureus* α-toxin [84] and has also been shown to mediate the effects of the antimicrobial compound taurine via mechanisms of both enhanced disease tolerance and resistance [85]. With regard to viral infections, during the central nervous system Sindbis Virus infection, autophagy exerts protective effects via tolerance mechanisms facilitating viral protein clearance and reducing virus-induced cell death, independent of direct effects on viral replication [86]. The predicted *miR-311-3p* targets *Ecdysone receptor* (*EcR*) [87,88], *Tumor protein p53 inducible nuclear protein* (*TP53INP*) [89], *blue cheese* (*bchs*) [90], *cacophony* (*cac*) [91], *Syntaxin 17* (*Syx17*) [92,93], and *serpent* (*srp*) [94] all possess validated roles as positive regulators of autophagy while *wacky* (*wcy*) acts as a negative regulator [95].

It is increasingly being recognized that metabolism plays a role in host immunity [96]. Using the oxygen consumption rate (OCR) as a proxy for metabolic rate readout, a higher OCR at 24 h post-infection was correlated with enhanced survival in FHV-infected flies at 48 h post-infection [97]. Another study supporting the importance of metabolism in disease tolerance employed an influenza model in mice, demonstrating that glucose was crucial to promoting disease tolerance by preventing initiation of endoplasmic reticulum (ER) stress-mediated apoptotic pathways [98]. Within our process and pathway analysis, metabolism-related terms were also prominently enriched across all groups of downregulated genes (Dataset S3). Among the plethora of predicted *miR-311-3p* targets with metabolic roles are the genes *Electron transfer flavoprotein-ubiquinone oxidoreductase* (*Etf-QO*), *NAD kinase* (*NADK*), *Carnitine palmitoyltransferase 2* (*CPT2*), and *Lipoic acid synthase* (*Las*).

Related to metabolism is reproduction, as there is a well-established trade-off between the resource-intensive and energetically costly processes of reproduction and immunity [50]. Supporting this trade off, employing *Drosophila* with germline removal found that these mutants experience greater expression of immune genes compared to controls both in the absence and presence of *Erwinia carotovora carotovora 15* (*Ecc15*) and *E. faecalis* infection [49]. In our process and pathway analysis of predicted targets of miRNAs upregulated only in young flies (Figure 2) and upregulated in both young and aged (Figure 4), reproduction-related terms were also significantly enriched.

There are various predicted *miR-311-3p* targets with validated roles in immune processes. Male *Drosophila* with loss of function of *boule* (*bol*) displayed defects in spermatogenesis due to disruption of entry into metaphase [99]. The gene *orb2* is also required during spermatogenesis to promote proper spermatid cyst polarization [100]. Other predicted *miR-311-3p* targets with suggested roles in reproduction based on expression patterns are *Polypeptide N-Acetylgalactosaminyltransferase 9* (*Pgant9*) [101], *Seminal fluid protein 77F* (*Sfp77F*) [102,103], *CG17271* [101], and *CG6168* [104]. Further investigation of the plethora of potential *miR-311-3p* gene targets could yield novel insights regarding the mechanisms underlying disease tolerance following viral infections.

While driving *miR-311* overexpression using *Act-5c-G4* resulted in developmental lethality, ubiquitous overexpression of another miRNA, *miR-284*, produced viable adults. Young *miR-284* OE males outlived genotypical controls following FHV indicating that this miRNA is sufficient to confer protection of FHV in this age group (Figure S11B). However, the fact that older *miR-284* OE flies displayed the opposite phenotype and died faster of FHV in comparison to controls (Figure S11C) suggests that a more complex age-dependent mechanism is in place that affects the outcomes of infection. Future studies could focus on evaluating the contribution of *miR-284* gene targets to the survival of FHV infection, as well as any potential sex-specific effects. To further evaluate miRNA function in aging antiviral immunity, additional experiments could be carried out with some of the candidate miRNAs that displayed comparable survival of FHV between young and aged flies in our study, or where aged flies outlived younger flies (e.g. *miR-989*, *miR-11*, *miR-10*, *miR-308*; Table 2). Finally, studies like the present work could be performed using other viruses that infect *Drosophila* to test if miRNAs identified here, including *miR-311*, participate more broadly in aging antiviral immunity.

## Supplementary Material

Suppl FigS3.jpg

Suppl FigS4.jpg

Suppl FigS7.jpg

Table S3.docx

Table S6.xlsx

Clean copy of supplementary material- QVIR-2024-0384.R1.docx

Suppl FigS8.jpg

Suppl FigS2.jpg

Table S5.docx

Suppl FigS10.jpg

Suppl FigS5.jpg

Suppl FigS1.jpg

Table S4.docx

Table S1.docx

Suppl FigS11.jpg

Suppl FigS12.jpg

Suppl FigS6.jpg

Suppl FigS9.jpg

Table S2.docx

## Data Availability

Raw Illumina miRNA sequence reads are available from the National Center for Biotechnology Information (NCBI) Sequence Read Archive (SRA) under BioProject PRJNA644593, accession numbers SRX23409571-SRX23409588. Supplemental material including Supplemental figures, tables, and datasets that support the findings of this study are openly available in the Open Science Framework (OSF) repository at https://osf.io/dzv7n/ (https://doi.org/10.17605/OSF.IO/DZV7N) [105].
