# Supplementary figures and images for: Investigating the differential microRNAs expression in young and aged Drosophila melanogaster following Flock House Virus infection
Source: Virulence. 2025 Aug 25;16(1):2549497. doi: 10.1080/21505594.2025.2549497 (PMC12380228; doi:10.1080/21505594.2025.2549497)

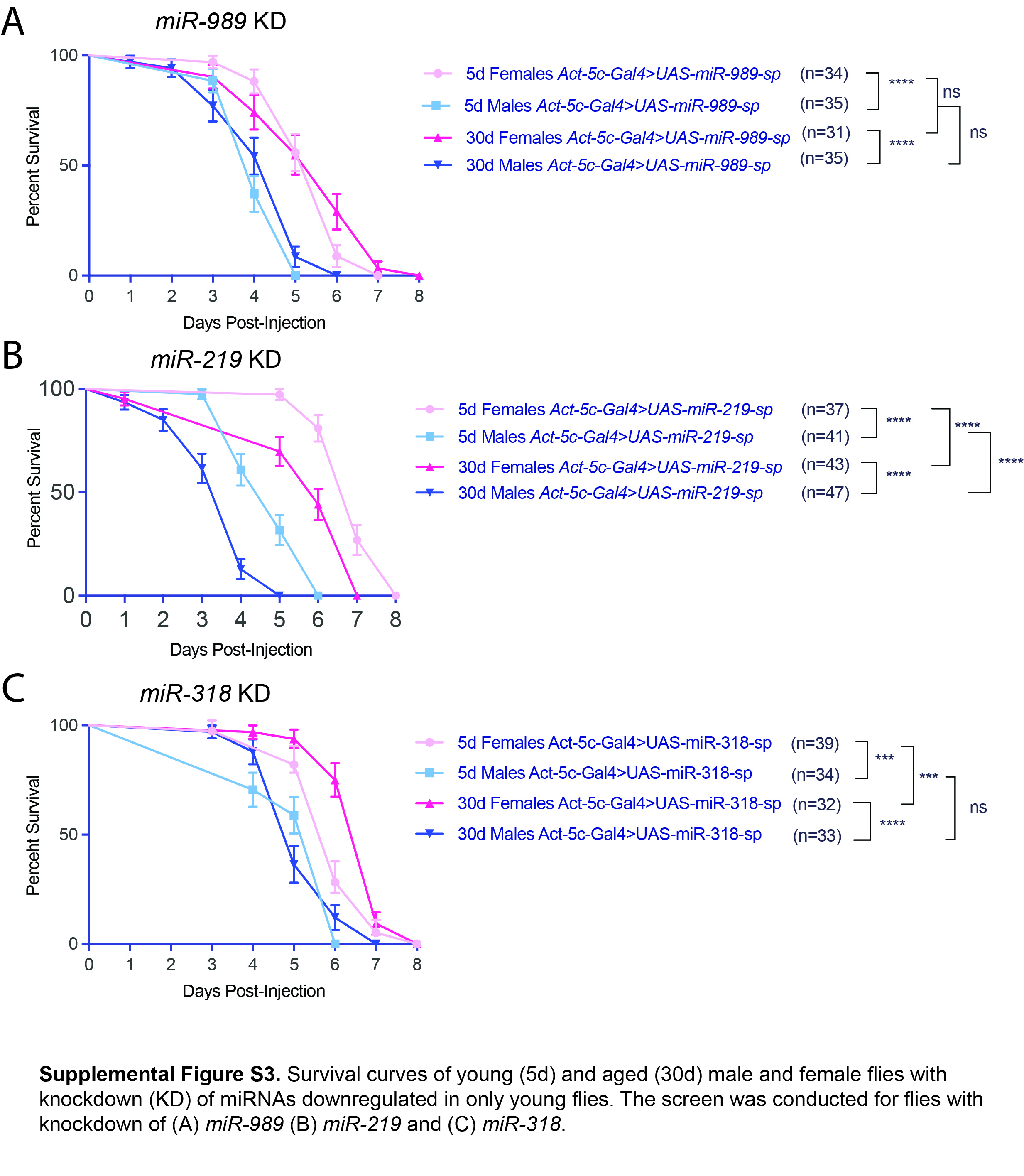

Supplement: Suppl FigS3.jpg [file KVIR_A_2549497_SM7102.jpg]

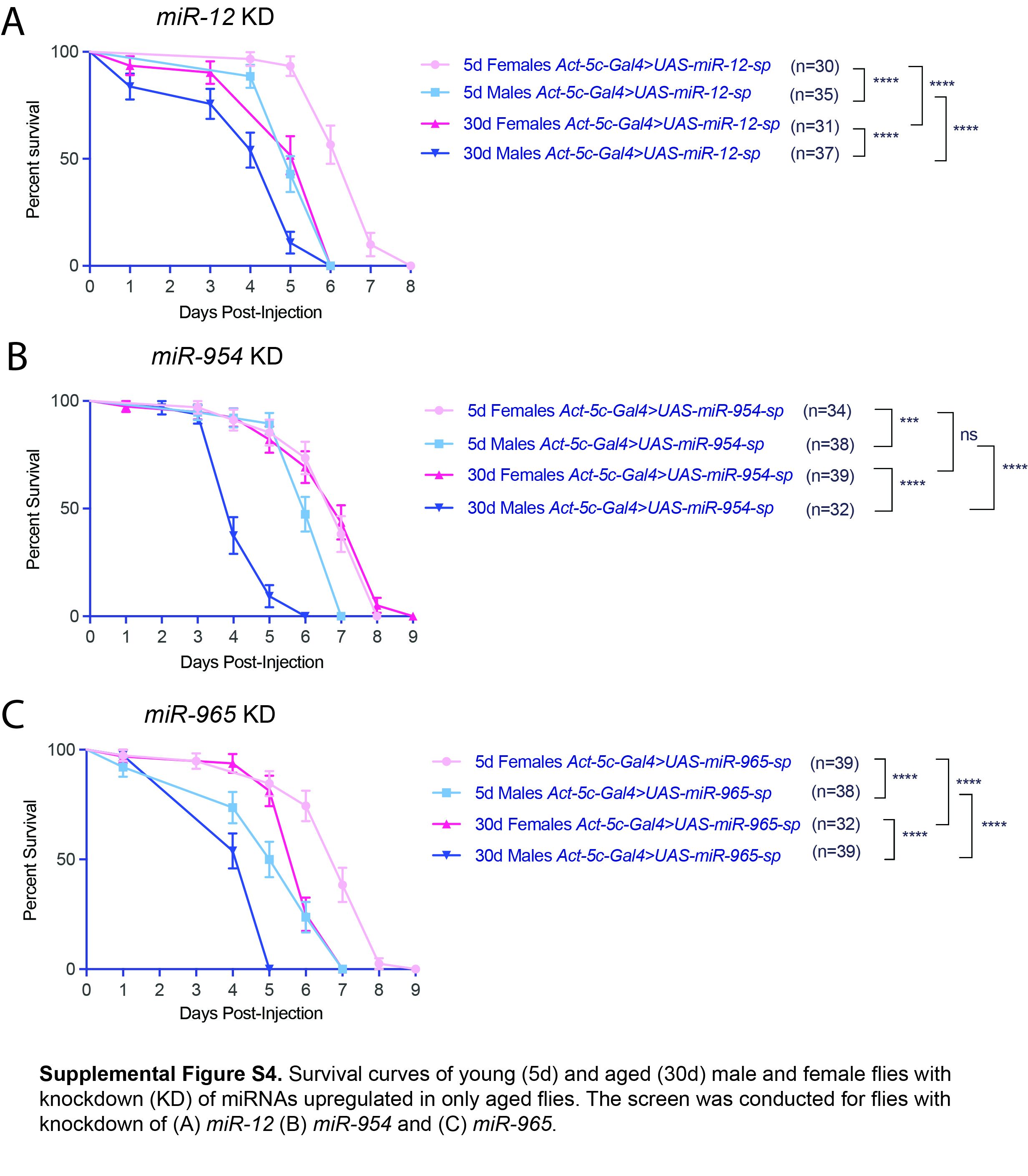

Supplement: Suppl FigS4.jpg [file KVIR_A_2549497_SM7101.jpg]

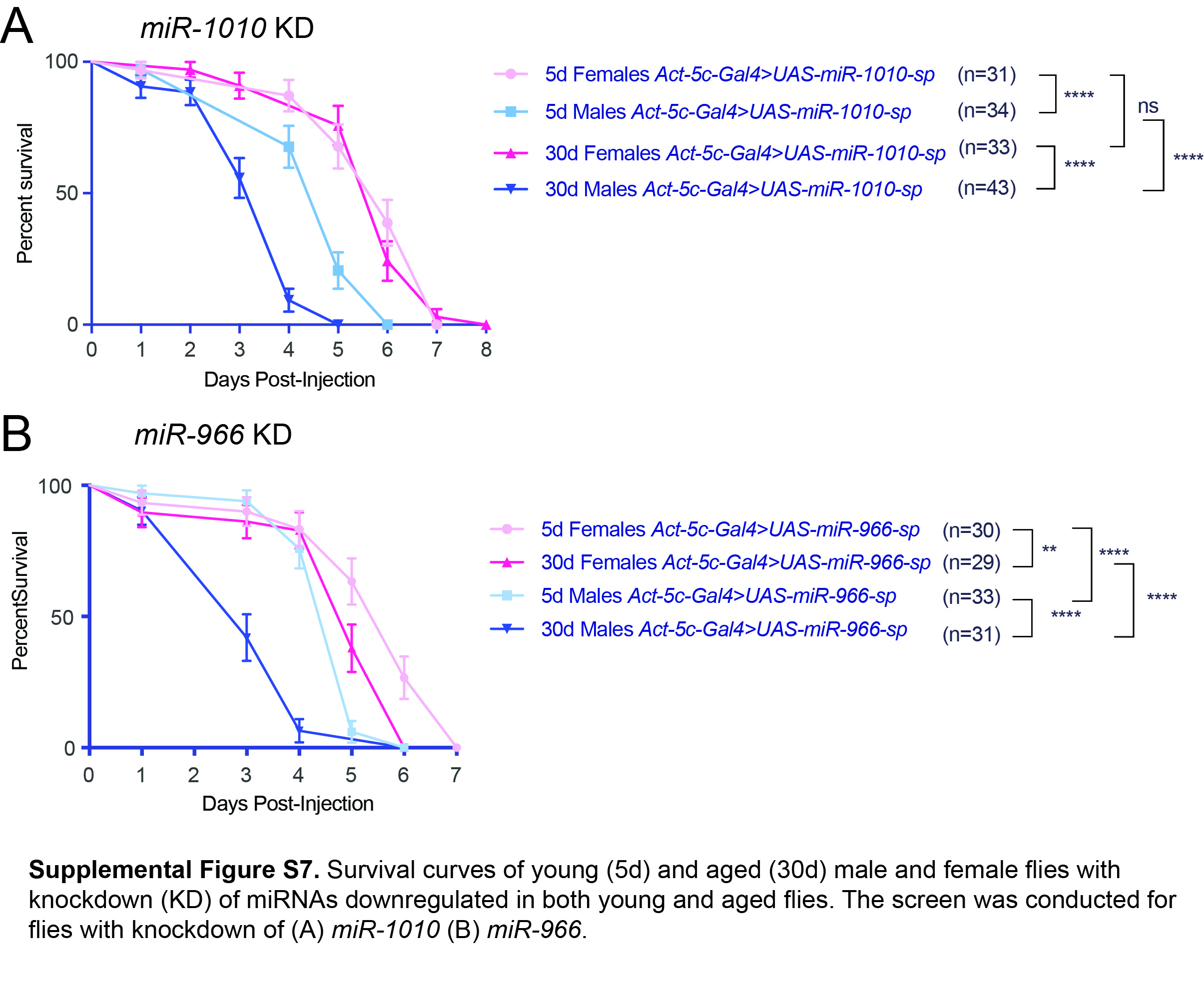

Supplement: Suppl FigS7.jpg [file KVIR_A_2549497_SM7100.jpg]

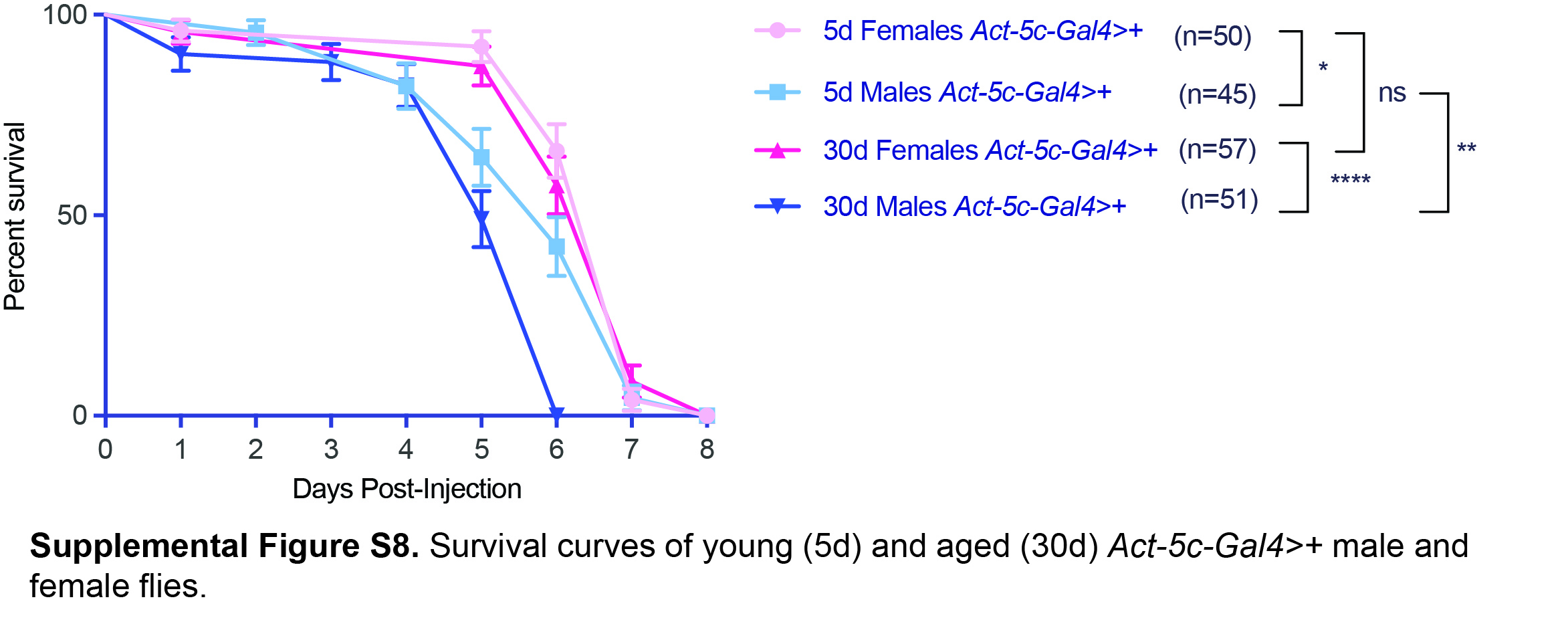

Supplement: Suppl FigS8.jpg [file KVIR_A_2549497_SM7096.jpg]

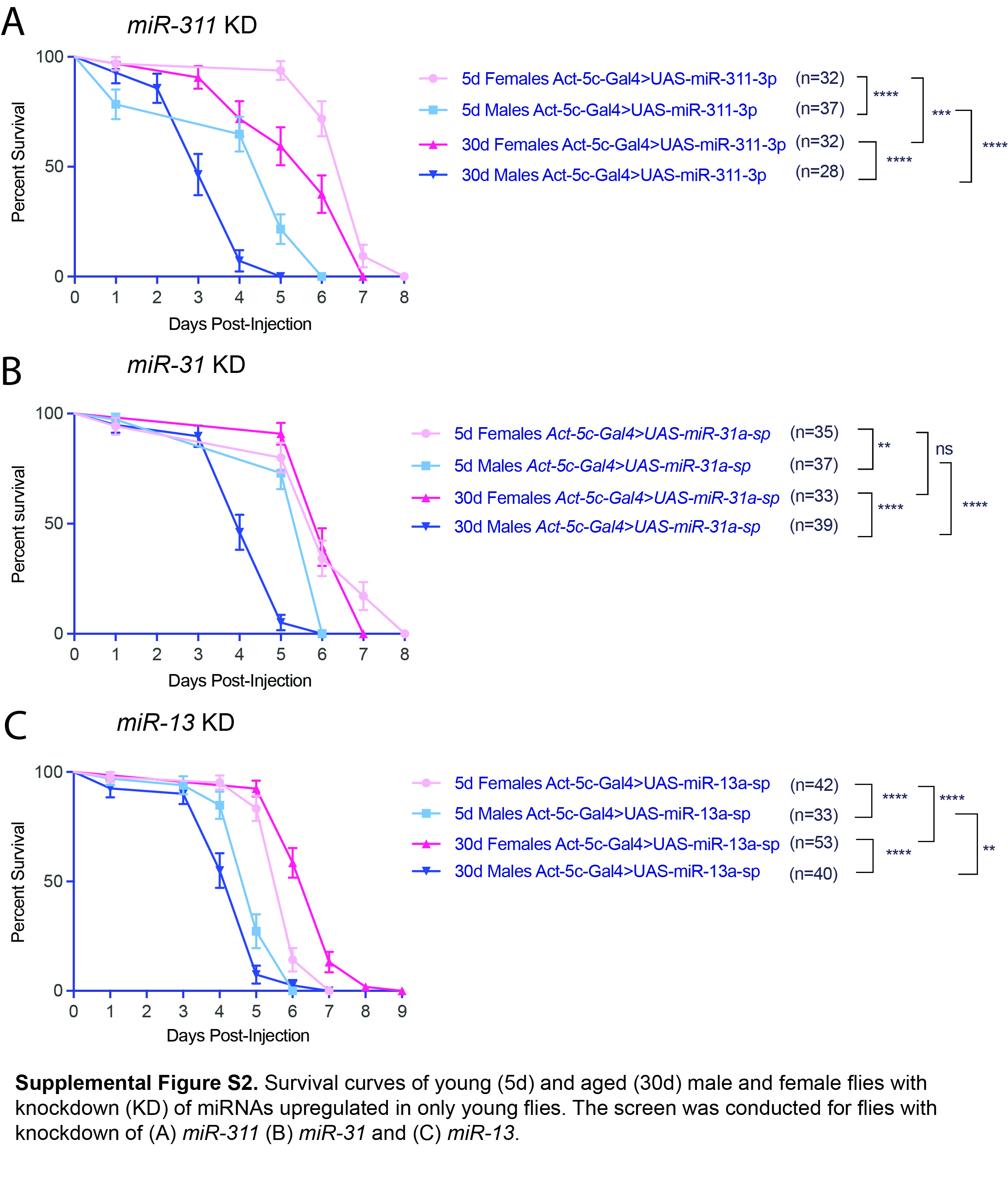

Supplement: Suppl FigS2.jpg [file KVIR_A_2549497_SM7095.jpg]

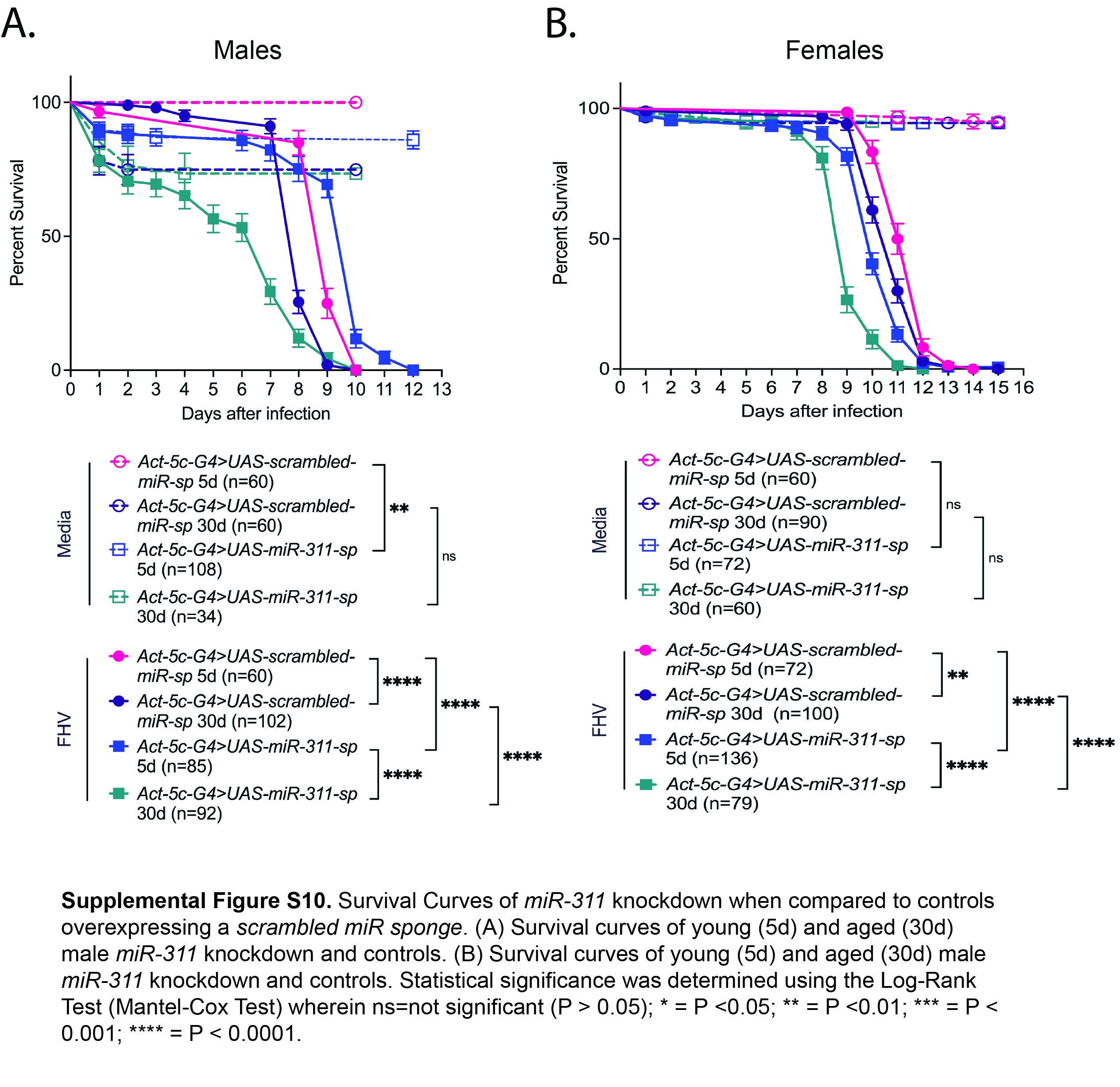

Supplement: Suppl FigS10.jpg [file KVIR_A_2549497_SM7093.jpg]

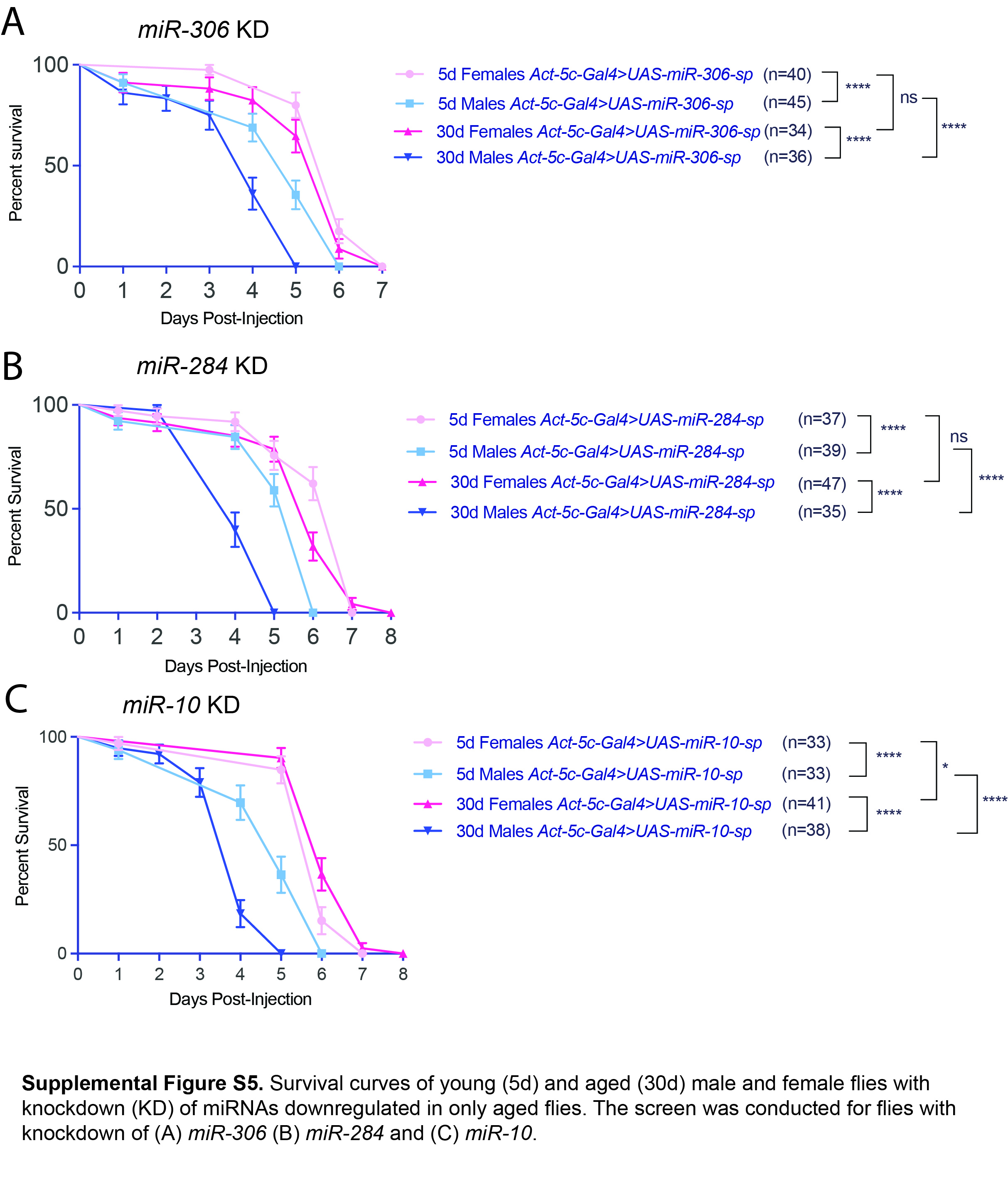

Supplement: Suppl FigS5.jpg [file KVIR_A_2549497_SM7092.jpg]

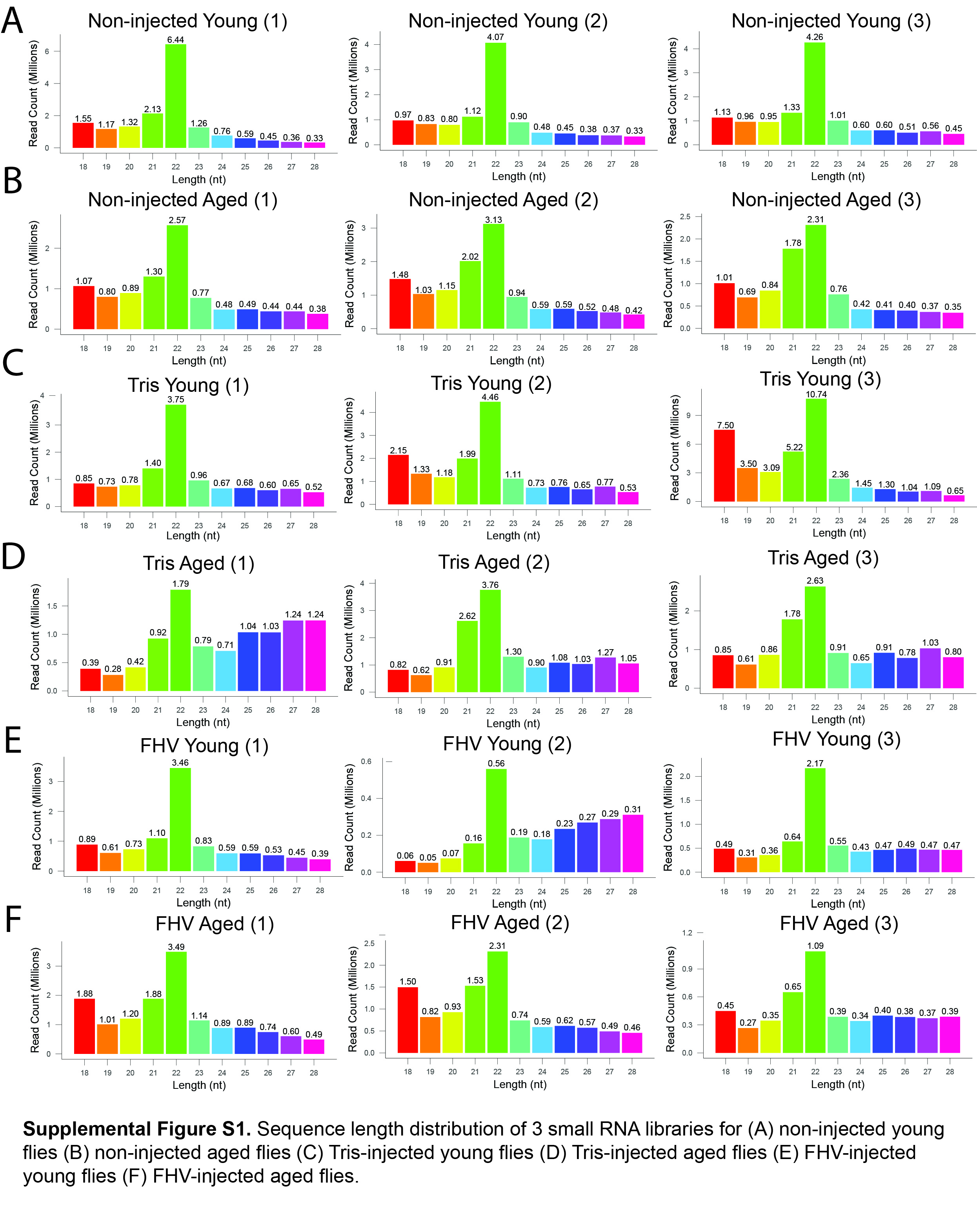

Supplement: Suppl FigS1.jpg [file KVIR_A_2549497_SM7091.jpg]

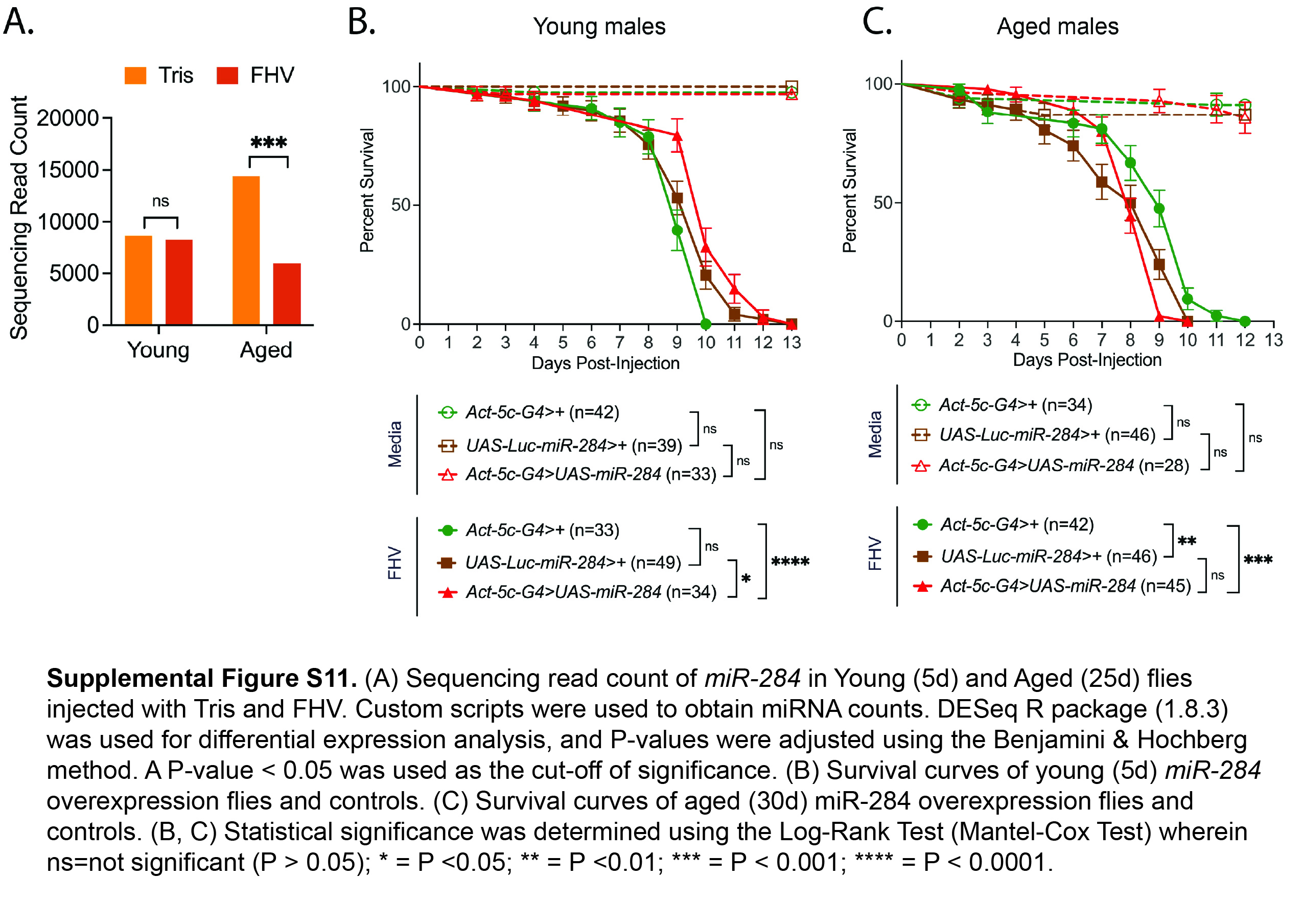

Supplement: Suppl FigS11.jpg [file KVIR_A_2549497_SM7088.jpg]

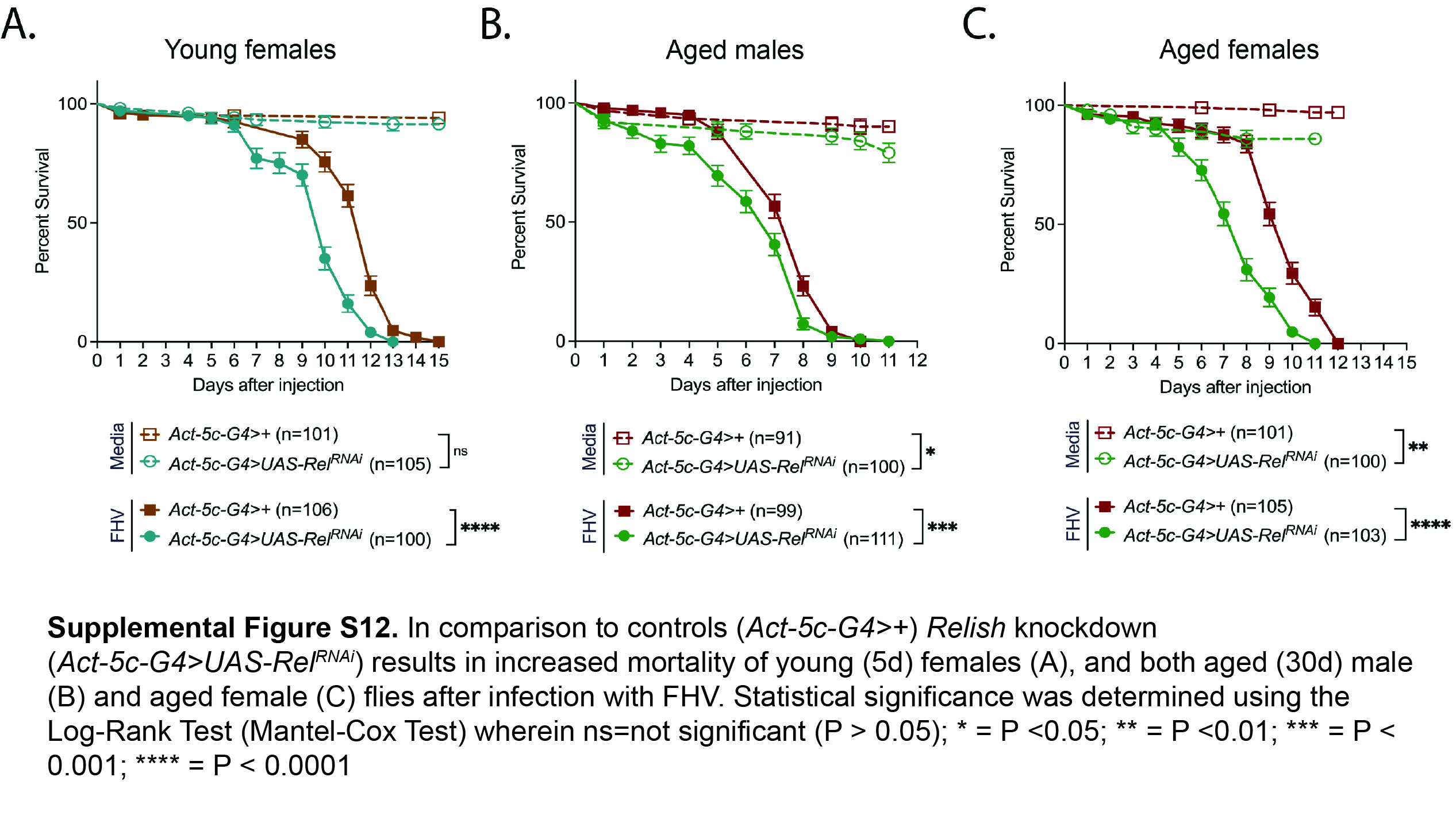

Supplement: Suppl FigS12.jpg [file KVIR_A_2549497_SM7087.jpg]

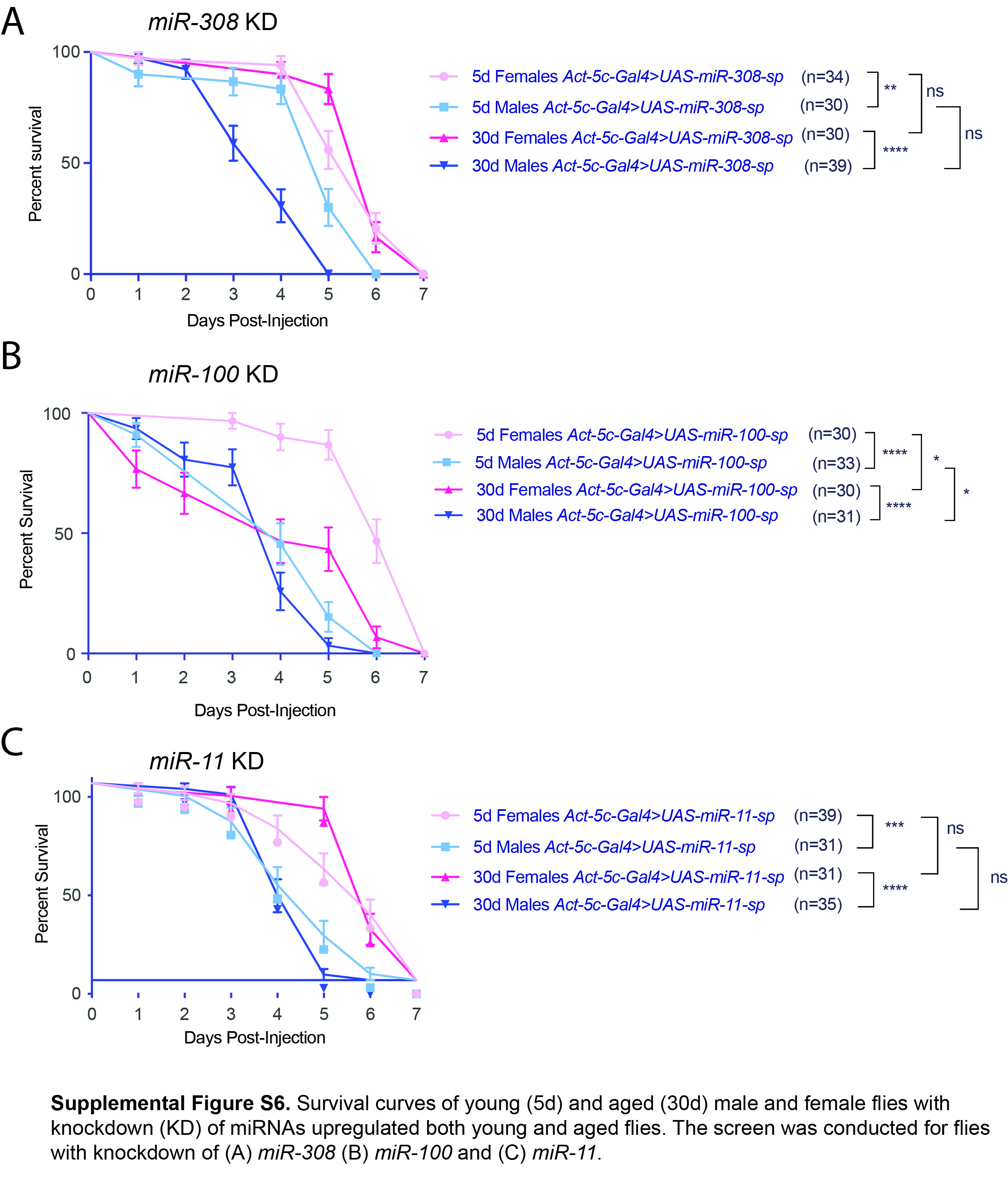

Supplement: Suppl FigS6.jpg [file KVIR_A_2549497_SM7086.jpg]

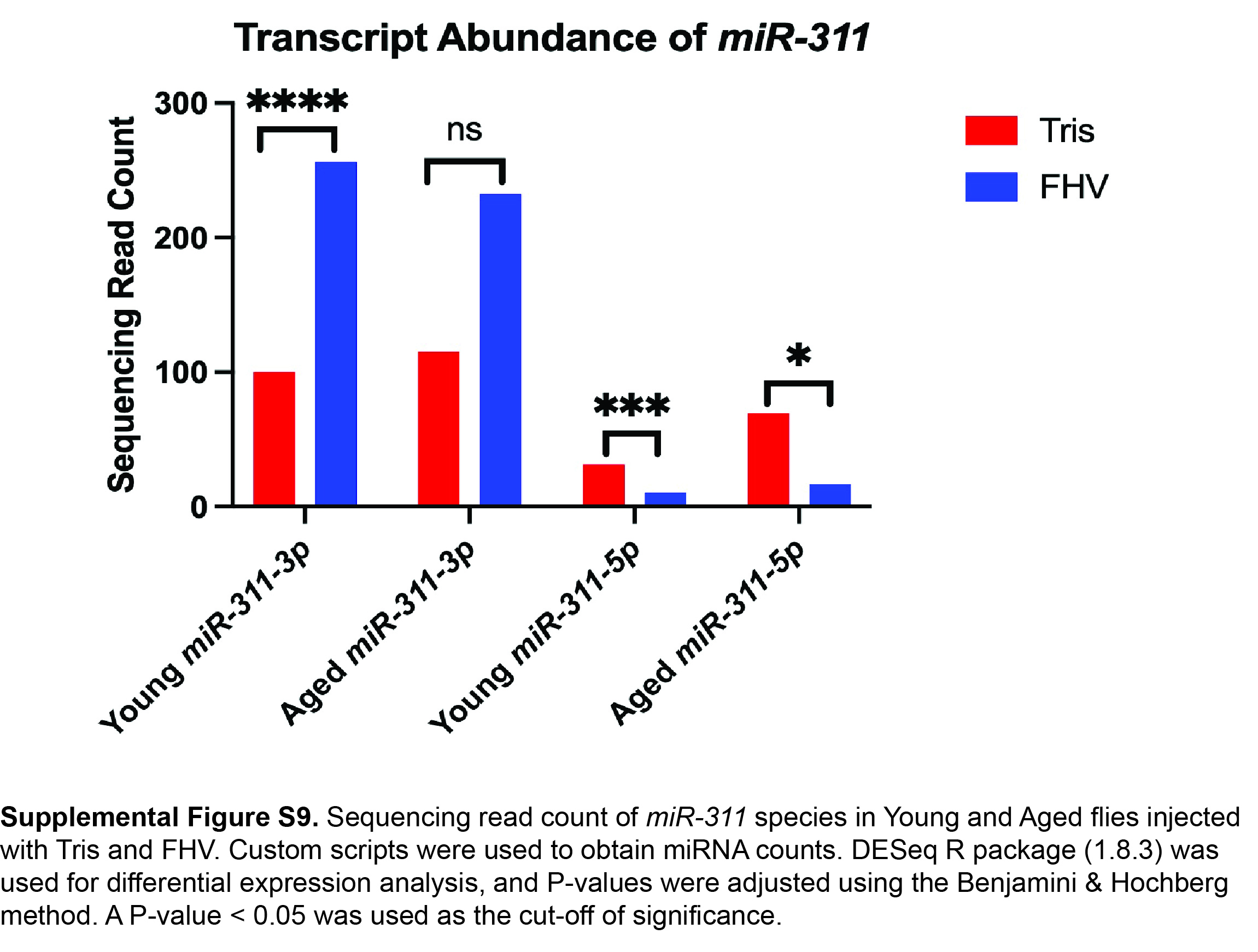

Supplement: Suppl FigS9.jpg [file KVIR_A_2549497_SM7085.jpg]
