# Supplementary material for: Investigating the differential microRNAs expression in young and aged Drosophila melanogaster following Flock House Virus infection
Source: Virulence. 2025 Aug 25;16(1):2549497. doi: 10.1080/21505594.2025.2549497 (PMC12380228; doi:10.1080/21505594.2025.2549497)
Supplement: Table S3.docx [file KVIR_A_2549497_SM7099.docx]

| Male vs Female | | | | |
| --- | --- | --- | --- | --- |
| miRNA Knocked Down | Young | | Aged | |
|  | Group which outlived | P-value (significance) | Group which outlived | P-value (significance) |
| None (*Act-5c-Gal4>+* control) | Females | 0.0144 (*) | Females | <0.0001 (****) |
| *mir-311* | Females | <0.0001 (****) | Females | <0.0001 (****) |
| *mir-31a* | Females | 0.0039 (**) | Females | <0.0001 (****) |
| *mir-13a* | Females | <0.0001 (****) | Females | <0.0001 (****) |
| *mir-989* | Females | <0.0001 (****) | Females | <0.0001 (****) |
| *mir-219* | Females | <0.0001 (****) | Females | <0.0001 (****) |
| *mir-318* | Females | 0.0003 (***) | Females | <0.0001 (****) |
| *mir-12* | Females | <0.0001 (****) | Females | <0.0001 (****) |
| *mir-954* | Females | 0.0004 (***) | Females | <0.0001 (****) |
| *mir-965* | Females | <0.0001 (****) | Females | <0.0001 (****) |
| *mir-306* | Females | <0.0001 (****) | Females | <0.0001 (****) |
| *mir-284* | Females | <0.0001 (****) | Females | <0.0001 (****) |
| *mir-10* | Females | <0.0001 (****) | Females | <0.0001 (****) |
| *mir-308* | Females | 0.0039 (**) | Females | <0.0001 (****) |
| *mir-100* | Females | <0.0001 (****) | Females | 0.0271 (*) |
| *mir-11* | Females | 0.0005 (***) | Females | <0.0001 (****) |
| *mir-1010* | Females | <0.0001 (****) | Females | <0.0001 (****) |
| *mir-966* | Females | <0.0001 (****) | Females | <0.0001 (****) |

**Table S3.** **Detailed display of survival differences among male and female miRNA KD flies to FHV infection**

All female miRNA KD lines exhibit enhanced survival to FHV infection compared to their age-

matched counterparts. Statistical significance was determined using the Log-Rank Test (Mantel-Cox Test) wherein ns=not significant (P > 0.05); * = P <0.05; ** = P <0.01; *** = P < 0.001; **** = P < 0.0001.
