# Supplementary material for: Investigating the differential microRNAs expression in young and aged Drosophila melanogaster following Flock House Virus infection
Source: Virulence. 2025 Aug 25;16(1):2549497. doi: 10.1080/21505594.2025.2549497 (PMC12380228; doi:10.1080/21505594.2025.2549497)
Supplement: Clean copy of supplementary material- QVIR-2024-0384.R1.docx [file KVIR_A_2549497_SM7097.docx]

**Supplemental figure legends**

**Supplemental Figure S1.**

Sequence length distribution of 3 small RNA libraries for (A) non-injected young flies (B) non-injected aged flies (C) Tris-injected young flies (D) Tris-injected aged flies (E) FHV-injected young flies (F) FHV-injected aged flies

**Supplemental Figure S2.**

Survival curves of young (5d) and aged (30d) male and female flies with knockdown (KD) of miRNAs upregulated in only young flies. The screen was conducted for flies with knockdown of (A) *miR-311* (B) *miR-31* and (C) *miR-13*

**Supplemental Figure S3.**

Survival curves of young (5d) and aged (30d) male and female flies with knockdown (KD) of miRNAs downregulated in only young flies. The screen was conducted for flies with knockdown of (A) *miR-989* (B) *miR-219* and (C) *miR-318*

**Supplemental Figure S4.**

Survival curves of young (5d) and aged (30d) male and female flies with knockdown (KD) of miRNAs upregulated in only aged flies. The screen was conducted for flies with knockdown of (A) *miR-12* (B) *miR-954* and (C) *miR-965*

**Supplemental Figure S5.**

Survival curves of young (5d) and aged (30d) male and female flies with knockdown (KD) of miRNAs downregulated in only aged flies. The screen was conducted for flies with knockdown of (A) *miR-306* (B) *miR-284* and (C) *miR-10*

**Supplemental Figure S6.**

Survival curves of young (5d) and aged (30d) male and female flies with knockdown (KD) of miRNAs upregulated both young and aged flies. The screen was conducted for flies with knockdown of (A) *miR-308* (B) *miR-100* and (C) *miR-11*

**Supplemental Figure S7.**

Survival curves of young (5d) and aged (30d) male and female flies with knockdown (KD) of miRNAs downregulated in both young and aged flies. The screen was conducted for flies with knockdown of (A) *miR-1010* (B) *miR-966*

**Supplemental Figure S8.**

Survival curves of young (5d) and aged (30d) *Act-5c-Gal4>+* male and female flies.

**Supplemental Figure S9.**

Sequencing read count of *miR-311* species in Young and Aged flies injected with Tris and FHV. Custom scripts were used to obtain miRNA counts. DESeq R package (1.8.3) was used for differential expression analysis, and P-values were adjusted using the Benjamini & Hochberg method. A P-value < 0.05 was used as the cut-off of significance

**Supplemental Figure S10.**

Survival Curves of *miR-311* knockdown when compared to controls overexpressing a scrambled miR sponge. (A) Survival curves of young (5d) and aged (30d) male *miR-311* knockdown and controls. (B) Survival curves of young (5d) and aged (30d) male *miR-311* knockdown and controls. Statistical significance was determined using the Log-Rank Test (Mantel-Cox Test) wherein ns=not significant (P > 0.05); * = P <0.05; ** = P <0.01; *** = P < 0.001; **** = P < 0.0001

**Supplemental Figure S11.**

(A) Sequencing read count of *miR-284* in Young (5d) and Aged (25d) flies injected with Tris and FHV. Custom scripts were used to obtain miRNA counts. DESeq R package (1.8.3) was used for differential expression analysis, and P-values were adjusted using the Benjamini & Hochberg method. A P-value < 0.05 was used as the cut-off of significance. (B) Survival curves of young (5d) *miR-284* overexpression flies and controls. (C) Survival curves of aged (30d) *miR-284* overexpression flies and controls. (B, C) Statistical significance was determined using the Log-Rank Test (Mantel-Cox Test) wherein ns=not significant (P > 0.05); * = P <0.05; ** = P <0.01; *** = P < 0.001; **** = P < 0.0001

**Supplemental Figure S12.**

In comparison to controls (*Act-5c-G4>+*) *Relish* knockdown (*Act-5c-G4>UAS-Rel^RNAi^*) results in increased mortality of young (5d) females (A), and both aged (30d) male (B) and aged female (C) flies after infection with FHV. Statistical significance was determined using the Log-Rank Test (Mantel-Cox Test) wherein ns=not significant (P > 0.05); * = P <0.05; ** = P <0.01; *** = P < 0.001; **** = P < 0.0001

**Supplemental tables legends**

**Supplemental Table S6.**

*miR-311-3p* Targets and Annotation.
