# Supplementary material for: Investigating the differential microRNAs expression in young and aged Drosophila melanogaster following Flock House Virus infection
Source: Virulence. 2025 Aug 25;16(1):2549497. doi: 10.1080/21505594.2025.2549497 (PMC12380228; doi:10.1080/21505594.2025.2549497)
Supplement: Table S5.docx [file KVIR_A_2549497_SM7094.docx]

| Temperature | Number of Desired Offspring (*Act-5c-Gal4; TubGal80^ts^>UAS-miR-311* lacking *Cyo* and *Tm6b* balancers) | | | Number of Undesired Offspring (containing *Cyo* and/or *Tm6b* balancers) | | | Percent of Desired Offspring | | |
| --- | --- | --- | --- | --- | --- | --- | --- | --- | --- |
|  | Replicate 1 | Replicate 2 | Replicate 3 | Replicate 1 | Replicate 2 | Replicate 3 | Replicate 1 | Replicate 2 | Replicate 3 |
| 18°C | 40 | 32 | 29 | 41 | 38 | 35 | 49.4% | 45.7% | 45.3% |
| 29°C | 0 | 0 | 0 | 19 | 16 | 17 | 0.0% | 0.0% | 0.0% |

**Table S5.** **Confirmation of Function *miR-311* Overexpression Construct**
