# Supplementary material for: Investigating the differential microRNAs expression in young and aged Drosophila melanogaster following Flock House Virus infection
Source: Virulence. 2025 Aug 25;16(1):2549497. doi: 10.1080/21505594.2025.2549497 (PMC12380228; doi:10.1080/21505594.2025.2549497)
Supplement: Table S4.docx [file KVIR_A_2549497_SM7090.docx]

| miRNA KD vs *Act-5c-Gal4>+* control | | | | |
| --- | --- | --- | --- | --- |
| miRNA Knocked Down | Males | | Female | |
|  | Group which outlived | P-value (significance) | Group which outlived | P-value (significance) |
| *mir-311* Young | Control | <0.0001 (****) | N/A | 0.3748 (ns) |
| *mir-311* Aged | Control | <0.0001 (****) | Control | 0.0079 (**) |
| *mir-31a* Young | Control | 0.0225 (*) | N/A | 0.1960 (ns) |
| *mir-31a* Aged | Control | <0.0001 (****) | N/A | 0.0966 (ns) |
| *mir-13a* Young | Control | 0.0001 (***) | Control | <0.0001 (****) |
| *mir-13a* Aged | Control | 0.0001 (***) | N/A | 0.4683 (ns) |
| *mir-989* Young | Control | <0.0001 (****) | Control | <0.0001 (****) |
| *mir-989* Aged | Control | <0.0001 (****) | Control | 0.0026 (**) |
| *mir-219* Young | Control | <0.0001 (****) | miRNA KD | 0.0040 (**) |
| *mir-219* Aged | Control | <0.0001 (****) | Control | 0.0446 (*) |
| *mir-318* Young | Control | 0.0022 (**) | Control | 0.0037 (**) |
| *mir-318* Aged | N/A | 0.7566 (ns) | N/A | 0.1944 (ns) |
| *mir-12* Young | Control | <0.0001 (****) | N/A | 0.8875 (ns) |
| *mir-12* Aged | Control | <0.0001 (****) | Control | <0.0001 (****) |
| *mir-954* Young | N/A | 0.4068 (ns) | miRNA KD | 0.0065 (**) |
| *mir-954* Aged | Control | <0.0001 (****) | miRNA KD | 0.0045 (**) |
| *mir-965* Young | Control | 0.0491 (*) | miRNA KD | 0.0037 (**) |
| *mir-965* Aged | Control | <0.0001 (****) | Control | 0.0047 (**) |
| *mir-306* Young | Control | <0.0001 (****) | Control | <0.0001 (****) |
| *mir-306* Aged | Control | <0.0001 (****) | Control | <0.0001 (****) |
| *mir-284* Young | Control | 0.0021 (**) | N/A | 0.3213 (ns) |
| *mir-284* Aged | Control | <0.0001 (****) | Control | 0.0202 (*) |
| *mir-10* Young | Control | <0.0001 (****) | Control | <0.0001 (****) |
| *mir-10* Aged | Control | <0.0001 (****) | N/A | 0.0841 (ns) |
| *mir-308* Young | Control | 0.00002 (***) | Control | <0.0001 (****) |
| *mir-308* Aged | Control | <0.0001 (****) | Control | 0.0012 (**) |
| *mir-100* Young | Control | <0.0001 (****) | N/A | 0.0617 (ns) |
| *mir-100* Aged | Control | <0.0001 (****) | Control | <0.0001 (****) |
| *mir-11* Young | Control | <0.0001 (****) | Control | 0.0002 (***) |
| *mir-11* Aged | Control | <0.0001 (****) | Control | 0.0097 (**) |
| *mir-1010* Young | Control | <0.0001 (****) | Control | 0.0039 (**) |
| *mir-1010* Aged | Control | <0.0001 (****) | Control | 0.0058 (**) |
| *mir-966* Young | Control | <0.0001 (****) | Control | 0.0001 (***) |
| *mir-966* Aged | Control | <0.0001 (****) | Control | <0.0001 (****) |

**Table S4.** **Detailed display of survival differences between miRNA KD flies and *Act-5c-Gal4>+* control to FHV infection**

miRNA KD in flies tends to impair survival to FHV infection compared to the *Act-5c-Gal4>+*

genotypical control, which is particularly prominent among young and aged male miRNA KD

lines. Statistical significance was determined using the Log-Rank Test (Mantel-Cox Test) wherein ns=not significant (P > 0.05); * = P <0.05; ** = P <0.01; *** = P < 0.001; **** = P < 0.0001.
