# Supplementary material for: Investigating the differential microRNAs expression in young and aged Drosophila melanogaster following Flock House Virus infection
Source: Virulence. 2025 Aug 25;16(1):2549497. doi: 10.1080/21505594.2025.2549497 (PMC12380228; doi:10.1080/21505594.2025.2549497)
Supplement: Table S1.docx [file KVIR_A_2549497_SM7089.docx]

| **Gene** | **Forward Primer (5’to 3’) Sequence** | **Reverse Primer (5’ to 3’) Sequence** |
| --- | --- | --- |
| *RpL32* | AAGAAGCGCACCAAGACTTCATC | TCTGTTGTCGATACCCTTGGGCTT |
| *FHV-1* | TTAGAGCACATGCGTCCAG | CGCTCACTTTCTTCGGGTTA |

**Table S1. qRT-PCR primer sequences**
