# Supplementary material for: Investigating the differential microRNAs expression in young and aged Drosophila melanogaster following Flock House Virus infection
Source: Virulence. 2025 Aug 25;16(1):2549497. doi: 10.1080/21505594.2025.2549497 (PMC12380228; doi:10.1080/21505594.2025.2549497)
Supplement: Table S2.docx [file KVIR_A_2549497_SM7084.docx]

| Young vs Aged | | | | |
| --- | --- | --- | --- | --- |
| miRNA Knocked Down | Males | | Females | |
|  | Group which outlived | P-value (significance) | Group which outlived | P-value (significance) |
| None (*Act-5c-Gal4>+* control) | Young | 0.0002 (***) | N/A | 0.6961 (ns) |
| *mir-311* | Young | <0.0001 (****) | Young | 0.0008 (***) |
| *mir-31a* | Young | <0.0001(****) | N/A | 0.6846 (ns) |
| *mir-13a* | Young | 0.0075 (**) | Aged | <0.0001 (****) |
| *mir-989* | N/A | 0.2302 (ns) | N/A | 0.3641 (ns) |
| *mir-219* | Young | <0.0001 (****) | Young | <0.0001 (****) |
| *mir-318* | Young | 0.9353 (ns) | Aged | 0.0009 (***) |
| *mir-12* | Young | <0.0001 (****) | Young | <0.0001 (****) |
| *mir-954* | Young | <0.0001 (****) | N/A | 0.6284 (ns) |
| *mir-965* | Young | <0.0001 (****) | Young | <0.0001 (****) |
| *mir-306* | Young | <0.0001 (****) | N/A | 0.0714 (ns) |
| *mir-284* | Young | <0.0001 (****) | N/A | 0.0907 (ns) |
| *mir-10* | Young | <0.0001 (****) | Aged | 0.0389 (*) |
| *mir-308* | Young | <0.0001 (****) | N/A | 0.3317 (ns) |
| *mir-100* | Young | 0.0352 (*) | Young | <0.0001 (****) |
| *mir-11* | N/A | 0.2798 (ns) | N/A | 0.4130 (ns) |
| *mir-1010* | Young | <0.0001 (****) | N/A | 0.7327 (ns) |
| *mir-966* | Young | <0.0001 (****) | Young | 0.0092 (**) |

**Table S2.** **Detailed display of survival differences among young and aged miRNA KD flies to FHV infection**

Young miRNA KD flies tend to exhibit enhanced survival of FHV infection compared to their

aged counterparts, especially amongst male miRNA KD flies. Statistical significance was determined using the Log-Rank Test (Mantel-Cox Test) wherein ns=not significant (P > 0.05); * = P <0.05; ** = P <0.01; *** = P < 0.001; **** = P < 0.0001.
